# Room Temperature Diels–Alder Reactions of 4-Vinylimidazoles

**DOI:** 10.3390/molecules29081902

**Published:** 2024-04-22

**Authors:** Brandon B. Fulton, Alexia J. Hartzell, H. V. Rasika Dias, Carl J. Lovely

**Affiliations:** Department of Chemistry and Biochemistry, University of Texas Arlington, Arlington, TX 76019, USA

**Keywords:** cycloaddition, heterocycle, alkaloids, intermolecular, intramolecular, DFT calculations

## Abstract

In the course of studying Diels–Alder reactions of 4-vinylimidazoles with *N*-phenylmaleimide, it was discovered that they engage in cycloaddition at room temperature to give high yields of the initial cycloadduct as a single stereoisomer. In certain cases, the product precipitated out of the reaction mixture and could be isolated by simple filtration, thereby avoiding issues with aromatization observed during chromatographic purification. Given these results, intramolecular variants using doubly activated dienophiles were also investigated at room temperature. Amides underwent cycloaddition at room temperature in modest yields, but the initial adducts were not isolable with *N*_imid_-benzyl-protected systems. Attempts to extend these results to the corresponding esters and hydroxamate were less successful with these substrates only undergoing cycloaddition at elevated temperatures in lower yields. Density functional theory calculations were performed to evaluate the putative transition states for both the inter- and intramolecular variants to rationalize experimental observations.

## 1. Introduction

**Figure 1:**
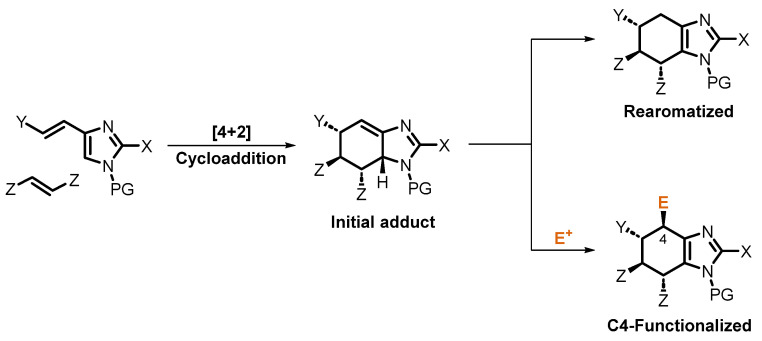
General reactivity of vinylimidazoles and their post-cycloaddition transformations.

The Diels–Alder reaction has a rich history in organic chemistry and has been widely studied from both a theoretical and synthetic perspective. The ability to create multiple rings and up to four stereocenters with high levels of relative and absolute stereochemical control is largely unparalleled in synthetic organic chemistry. As a result, investigations describing new applications of this venerable transformation continue to emerge in the literature [1,2,3,4,5,6,7,8]. Not unexpectedly, this reaction has been applied to the synthesis of both simple and complex heterocyclic molecules [9] and, in particular, vinyl heterocycles [10] have proven to be useful in synthesis [11,12,13,14,15,16,17,18,19,20,21,22,23,24]. Our lab has explored the chemistry of 4-vinylimidazoles extensively and has reported both inter- and intramolecular variants for the construction of polysubstituted tetrahydro- and dihydrobenzimidazoles; [25,26,27,28,29,30,31,32,33,34] these investigations continue. Reactions proceed with generally high *endo* selectivity and produce either the initial adduct or the rearomatized adduct depending on the nature of the substrate and the reaction conditions (Figure 1). The initial adducts are valuable as it has been shown by our lab [27,35] and others that the double bond is sufficiently reactive to undergo addition with electrophiles (or enophiles) to provide highly elaborated adducts in good yields and stereoselectivities (Figure 1) [20]. Our initial interest in this chemistry was driven by its use in approaches to a number of natural product targets [32,36], particularly dimeric members of the oroidin family such as ageliferin, axinellamine, massadine and palau’amine [37,38].

## 2. Results and Discussion

### 2.1. Chemistry

**Scheme 1:**
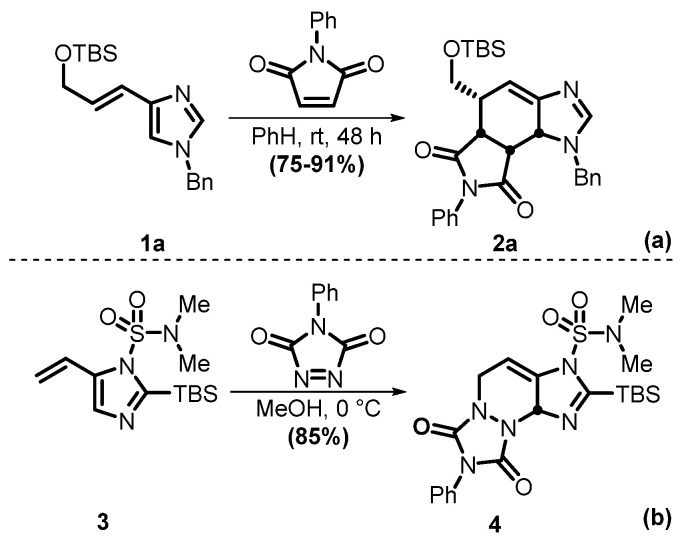
Examples of room temperature or below Diels–Alder reactions of vinylimidazoles (**a**) The first observation from our group. (**b**) Prior literature precedent.

In all of these investigations, despite observing high diastereoselectivities, our lab has never before devoted much attention to investigating asymmetric variants of these reactions, even though the utility of such reactions is obvious. As part of an effort to identify strategies to develop asymmetric versions of this reaction, initial control reactions were performed to demonstrate that there was no background reaction at room temperature. Our initial hypothesis was that overcoming the dearomatization barrier, although relatively low compared to benzene, would require some level of thermal activation. Exploratory experiments we had conducted when this reaction was first being investigated were performed in various solvents at reflux with temperatures as low as ca. 40 °C (CH_2_Cl_2_), but no lower [25]. A typical procedure involved running the reaction in a sealed tube with degassed solvents and isolating the product by either chromatography or, in some cases, cooling to room temperature, concentrating the reaction mixture to approximately 50% by rotary evaporation, and collecting the precipitated product by filtration. Our previously reported method using the Bn (benzyl)-protected vinylimidazole **1a** at a concentration of 0.1 M in degassed benzene with *N*-phenylmaleimide (NPM) as the dienophile partner led to a 76% yield when conducted at 55 °C for 24 h using this latter procedure [28]. Upon reexamination of this reaction, an attempted control reaction was conducted using the same concentrations at room temperature with only trace amounts of the cycloadduct being observed after 48 h. However, as a means of bypassing the concentration step required for purification, it was thought that increasing the concentration of the reaction mixture might be successful. Accordingly, when the reaction of NPM was performed for 48 h at room temperature with an increased concentration (0.125 M), cycloadduct **2a** gradually precipitated from the reaction mixture and could be collected by simple filtration, leading to a moderate yield of 48%. This yield could be increased to 75% by again halving the volume of the filtrate to promote precipitation (Scheme 1a). Extension of the reaction time (96 h) and recrystallization from the filtrate to yielded a second crop which resulted in an increased yield (83%). This variant was conducted on multigram scales (>20.0 g of **1a**). Subsequent studies showed that increasing the concentration even more both reduces reaction time and increases the yield, with a 21.0 g reaction of **1a** at a concentration of 0.5 M, leading to 91% yield after only 48 h. This reaction can also be performed in toluene, though slightly reduced yields (78%) are obtained as NPM is slightly less soluble in toluene and thus harder to wash away during filtration. It is hypothesized that the precipitation, which occurs during the reaction, retards either the rearomatization process or potential retro-Diels–Alder reactions. A similar example of vinylimidazoles participating in Diels–Alder reactions was reported by Koomen and co-workers, featuring 5-vinylimidazole **3** undergoing cycloaddition with 4-phenyl-1,2,4-triazoline-3,5-dione at 0 °C to produce cycloadduct **4** in 85% yield (Scheme 1b) [21]. There are several features of this reported reaction worth mentioning. First, the reaction involving **3** also resulted in the initial cycloadduct precipitating out and thus benefits from the same presumed protections against rearomatization as our reaction. In addition, the electron withdrawing sulfonylurea protecting group likely makes the cycloadduct less prone to rearomatization through the delocalization of the nitrogen lone pair, and the dienophile used is more reactive, thus allowing potentially even lower reaction temperatures (0 °C), which presumably favors product crystallization.

**Scheme 2:**
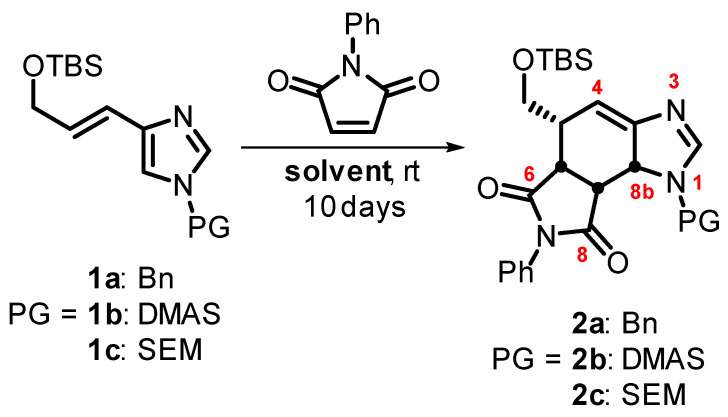
Room temperature Diels–Alder reactions of 4-vinylimidazoles.

**Figure 2:**
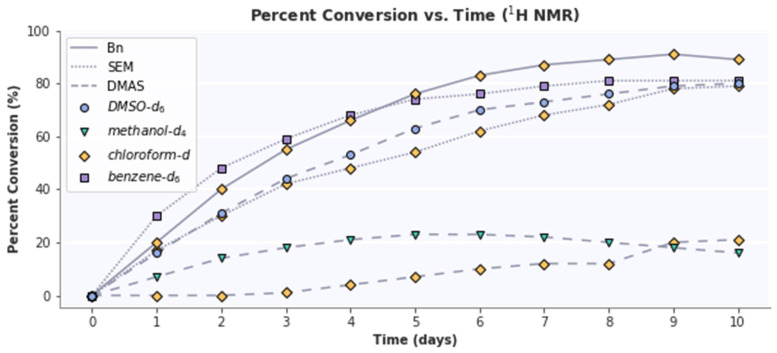
Room temperature Diels–Alder reactions of vinylimidazoles **1a**–**c** with NPM. Yields monitored by NMR spectroscopy.

**Figure 3:**
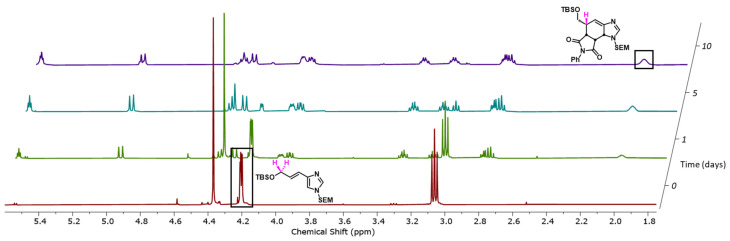
Time course study of the Diels–Alder reaction between SEM-protected vinylimidazole **1c** and NPM 0, 1, 5, and 10 days monitored by ^1^H NMR spectroscopy. Boxes indicate the peaks used for integration with the corresponding protons highlighted in pink.

Given our initial observations and those by the Koomen lab, it became of interest to determine whether these examples were simply limiting cases or whether these room temperature (or below) cycloadditions were more broadly applicable with vinylimidazoles. Over the years, we have developed syntheses of a number of variously protected vinylimidazoles to investigate the impact of the imidazole protecting group on reactivity. In particular, those containing moderately or even strongly electron-withdrawing substituents were of interest. Initial room temperature experiments were conducted with three different vinylimidazoles (PG = Bn **1a**, dimethyl aminosulfonyl (DMAS) **1b**, and 2-(trimethylsilyl)ethoxymethyl (SEM) **1c**) in benzene-d_6_ and the reaction progress was monitored by acquiring the ^1^H NMR spectra approximately every 24 h over the course of 10 days (see Scheme 2, Table 1, Figure 2 and Figure 3 and Supplementary Materials). All three substrates engaged in Diels–Alder reaction with *N*-phenylmaleimide with the appearance of a cycloadduct after 24 h. For the SEM-derivative **1c**, the product **2c** intensity increased steadily over the 10 day period, reaching a maximum at the 8-day mark. After the initial observation of cycloadduct in the NMR spectrum for the Bn- and DMAS-protected congeners, the solution became cloudy as a result of the cycloadduct precipitating out. To obtain a better idea of the intrinsic reactivity of the Bn- and DMAS-protected congeners, the reactions were repeated in other solvents. In deuteriochloroform (the chloroform was freed from acidic residues by passing it through a short pad of alumina prior to use), the Bn derivative **1a** produced the cycloadduct **2a** over the 10-day observation period up to ca. 84% completion, whereas the DMAS derivative **1b** provided the cycloadduct **2b** but only at ca. 20% conversion. Accordingly, the cycloadditions of **1b** were repeated in perdeuterated methanol and DMSO; in methanol, the reaction reached the same levels of conversion (ca. 20%) as chloroform but stalled after five days. On the other hand, in DMSO, the reaction was much more efficient, proceeding up to approximately 70%. It should be noted that the DMAS-protected derivative **1b** affords the initial adduct **2b** under thermal activation very nicely in either benzene or dichloromethane [25,28].

**Scheme 3:**
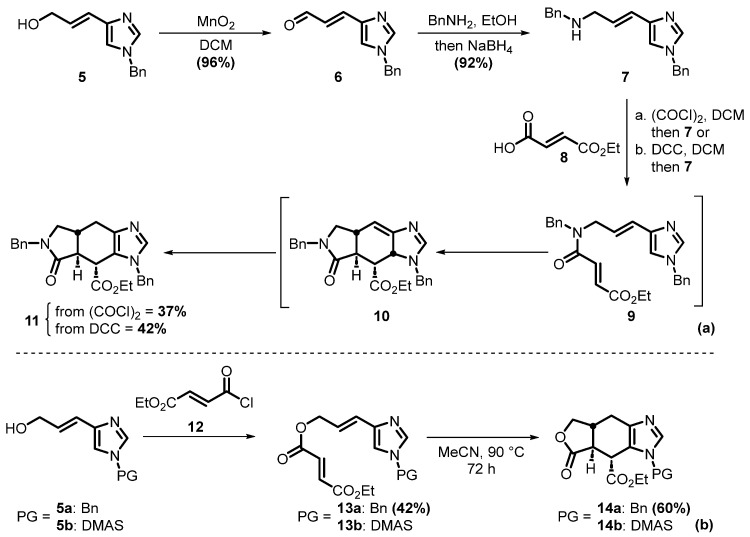
Preparation and cyclization of fumaramide and fumarate derivatives.

Given these observations with intermolecular reactions, we were curious whether intramolecular variants with doubly activated dienophiles might also proceed at room temperature. Such substrates were of interest because several synthetic projects would benefit from access to the initial adduct as it provides a gateway to further functionalization and potential application to syntheses of several oroidin dimers. Prior investigations with pseudodimeric substrates delivered aromatized cycloadducts under thermal conditions in good yields and stereoselectivities, but the elevated temperatures required for successful cycloaddition precluded the isolation of the initial adducts [26,29,30,31,32]. Early investigations with amido systems suggested that fumaramide derivatives may be of interest as we had noted (during their preparation) a small amount of cycloaddition [26,31], although at that time, we had attributed it to heating (ca. 40 °C) during the rotary evaporation of the solvent after extractive workup. At the time of this initial investigation, we had not prepared the *N*_imid_-Bn derivative and examined its cycloaddition. To prepare this substrate, the known *N*-benzyl amine **7** was prepared by oxidation of the corresponding alcohol **5** and a two-step/one-pot reductive amination sequence (Scheme 3a) [26,31]. Acylation with the mono acid chloride of fumaric acid provides the cycloaddition precursor **9**. Attempts to isolate **9** proved futile as the cycloaddition was very rapid and led directly to a mixture of the initial cycloadduct **10** and the aromatized derivative **11** [39]. We were unable to isolate the initial adduct due to in situ aromatization; this type of rearomatization had been observed previously with the DMAS-protected congener if it was not purified prior to submission to Diels–Alder conditions. Presumably, traces of acid catalyze the rearomatization process. Repetition of the synthesis using a DCC coupling of the acid **8** and the amine yields the precursor **9** (observed by ^1^H NMR spectroscopy of the crude reaction mixture) but results in the formation of the aromatized adduct after silica gel chromatography. Attempts were made to oxidize the intermediate cycloadduct **10** with DMDO and MCBPA to afford the corresponding alcohol, conditions that work well in other cases, but only very small amounts of the alcohol formed which was difficult to purify.

**Figure 4:**
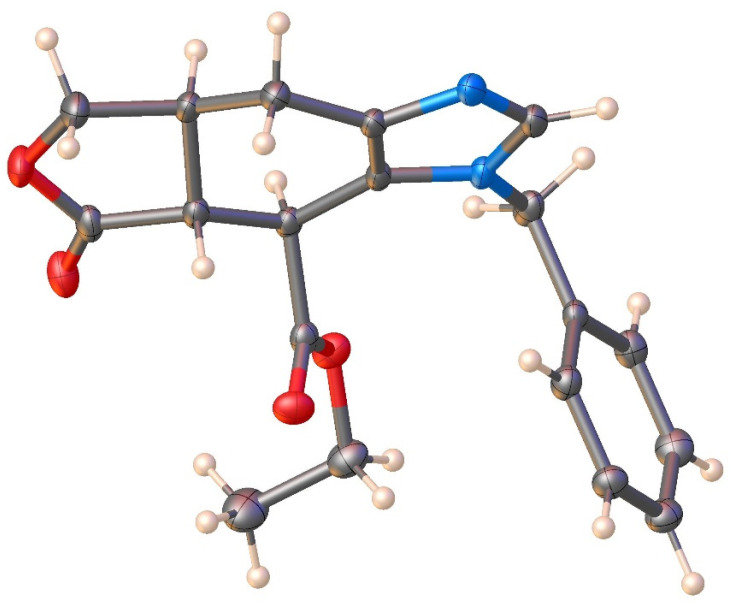
X-ray crystal structure of compound **14a** (CCDC 2327107).

The *N*-Bn amides are excellent cyclization substrates due to the amide *N*-substituent enhancing the population of the reactive rotamer, and so, it was no surprise that these substrates engaged in cycloaddition [39]. On the other hand, while the corresponding fumarates are a little easier to prepare, they tend to be less reactive because they do not benefit from enhanced reactive rotamer populations. However, we were curious to establish whether they would engage in cycloaddition under appropriate milder conditions than previously reported [26,31]. Accordingly, both Bn- and DMAS-protected congeners **13a**–**b** were prepared from the corresponding alcohols as before (Scheme 3b). We have previously described some cycloaddition reactions of **13a**–**b** at elevated temperatures and found the sulfonyl urea **13b** to be poorly reactive, whereas **13a** behaved better in benzene [26,31]. However, attempts to engage the Bn-protected **13a** analog in cycloaddition at room temperature in deuterated benzene, DMSO, and acetonitrile were unsuccessful. We then explored elevated temperatures starting with benzene, which showed no reaction under 100 °C. As previous examples with such elevated temperatures have solely led to rearomatized products, this solvent was abandoned, and we turned to other options. One thought for reducing the temperature for this intramolecular Diels-Alder reaction, even if room temperature was not an option, was to investigate solvents that mitigate the increased molecular dipole that develops in transition states of cycloaddition reactions of esters. It has been shown by Gervay and Jung that solvent polarity can play a significant role in the intramolecular cycloaddition reactions of fumarate esters, specifically in the case of acetonitrile and DMSO [40,41]. Accordingly, we examined both these solvents and found some reduction in reaction temperature, but were still unable to isolate the initial cycloadducts. For the case of DMSO-d_6_, a conversion of less than 10% was observed after 12 h at 40 °C. Increasing the temperature to 80 °C for 8 h led to a slightly increased rate with a yield of approximately 25%. However, no peaks for the initial adduct were observed in the ^1^H NMR spectrum and attempts to remove the product from DMSO using extractive workups led to significantly decreased yields. Attempts with acetonitrile-d_3_ commenced by monitoring the reaction at room temperature for 5 days, with no formation of cycloadducts being observed. Increasing the temperature to 45 °C, again for 5 days, led to the formation of aromatized product while no enamine peak was observed in the ^1^H NMR spectrum. For characterization of the aromatized product, the reaction was run at 90 °C for 72 h in acetonitrile, resulting in a 60% yield of the major product, which was confirmed as the *endo* adduct by X-ray crystallography (Figure 4, see also Supplementary Materials Figure S1, Tables S3–S5). Disappointingly, none of the esters underwent low-temperature cycloaddition, nor did they provide the initial adduct.

**Scheme 4:**
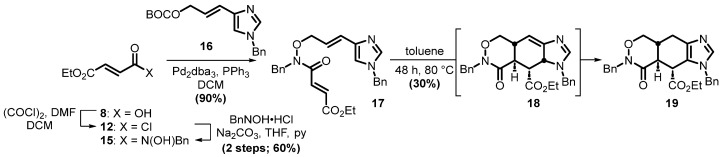
Preparation and cyclization of hydroxamate-linked derivatives.

In previous studies directed towards the oroidin dimers, axinellamine and massadine [29], we examined the use of hydroxamates as linking units between the diene and dienophile components as they provide the opportunity for cleavage under relatively mild reductive conditions (e.g., SmI_2_, Zn/HOAc) [42]. In these studies, we only examined pseudodimers, i.e., where the dienophile also contains an imidazole; and thus, we took this opportunity to examine the fumarate-derived system. The corresponding half-hydroxamic acid **15** was prepared according to the procedure from [43], and then using Tsuji–Trost chemistry was coupled with the *t*-butyl carbonate **16** derivative, affording the cycloaddition precursor **17** in excellent yield (Scheme 4) [29,44]. Unlike the amide substrate, the hydroxamate could be isolated by column chromatography and fully characterized. However, the Diels–Alder reaction of the hydroxamate proved to be stubborn and required elevated temperatures and often led to inseparable mixtures of products. Characterization of the cycloadduct was obtained by running the reaction in toluene for 2 days at 80 °C, leading to a low 30% yield of predominately the *endo* adduct. Even after column chromatography, traces of impurities remained—possibly the *exo* Diels–Alder adduct.

### 2.2. DFT Investigation

**Figure 5:**
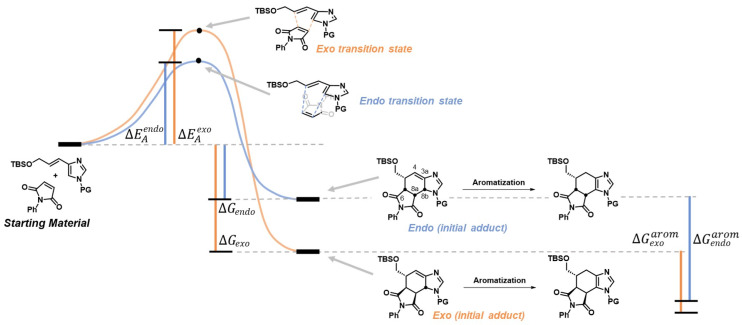
DFT study of Diels–Alder reactions of 4-vinylimidazoles. Energy values calculated for starting materials, *endo* and *exo* transition states, their corresponding initial cycloaddition adducts, and their corresponding rearomatized products.

As noted previously, there was initially some surprise that several of these cycloadditions proceeded at room temperature, and therefore, we were curious whether density functional theory (DFT) calculations might provide insight concerning the kinetic barriers to these reactions, the high diastereoselectivities observed, and the relative stabilities of the two adducts (initial vs. rearomatized). To probe these questions, DFT energy calculations were conducted for starting materials, *endo* and *exo* transition states (Supplementary Materials, Table S1), the corresponding initial prearomatic Diels–Alder adducts, and their aromatized tetrahydrobenzimidazole counterparts (Figure 5, Table 2, Supplementary Materials, Table S2). Both the aforementioned inter- and intramolecular variants were assessed along with the incorporation of solvent modeling for the solvents used in the NMR time course studies. Details of the computational methods are given in the Experimental Section in the Supplementary Materials’ information.

Inspection of the resulting data revealed several features warranting further discussion. A comparison of the energies for the *endo* and *exo* transition states (Table 2) shows that the *exo* adduct is higher in energy by ≈ 2.5–5.0 kcal/mol for all intermolecular cases, while the transition states for the intramolecular variants show a lower preference for *endo* over *exo* pathways with several cases favoring *exo* by ≈ 1.0 kcal/mol when solvent models are used (Table 2, entry 5). The results for the intermolecular reactions are supported by the experimental observation, wherein all of the room temperature Diels–Alder reactions with NPM provided *endo* isomers in good yield (>70%). For the intramolecular cases, the isolated products are derived from an *endo*-derived transition state (the CO_2_Et in an *endo* location). However, these cycloadditions, with the exception of the amides, do not occur at room temperature and are lower yielding in general. As a result, the initial adducts are not readily isolable due to the increased reaction temperatures required, which facilitates rearomatization.

Another aspect we wanted to probe was the stability gained from the rearomatization process (Table 3). To explain prior experimental work in which we were able to isolate the initial non-aromatized Diels–Alder adducts, we had qualitatively rationalized these observations on the basis that the products were stabilized by conjugation of the 3a,4-bond with the imidazole C=N bond. As expected, our energy calculations revealed that for all systems, both inter- and intramolecular, the aromatic congeners are 15–25 kcal/mol more stable, suggesting that the rearomatization process is highly thermodynamically favorable, with intramolecular cases having 5–10 kcal/mol more stabilization than the intermolecular cases. As we were able to isolate the prearomatic products for reactions conducted at lower temperatures, this implies that there is likely a substantial kinetic barrier to rearomatization. Additionally, rearomatization can be prevented experimentally by avoiding high temperatures or acidic conditions. This rearomatization formally occurs though a net [1,3]-H shift, but this process is thermally forbidden, at least via a geometrically accessible concerted antarafacial transition state. Presumably, therefore, rearomatization must occur via a proton transfer pathway, although in certain cases a radical pathway has been postulated [45]. Although we have no direct evidence for the mechanism [33,34,46], our assumption is that it most likely occurs through C4 protonation followed by deprotonation of C8b. In this manifold, it is not clear what the proton source is or what the proton acceptor is for the second step. In principle, it could occur the other way around, but it is assumed that the proton at C8b is insufficiently acidic under the reaction conditions and it is somewhat sterically encumbered. The precise details notwithstanding, this pathway is not kinetically viable at room temperature or even at elevated temperatures in some cases, which allows for the isolation of the initial adducts **2a**–**c**. Broadly speaking, the relative stability of the initial adducts mirror our expectations with SEM- and DMAS-protected congeners exhibiting higher stability than the Bn derivative, which has been interpreted in terms of the electron-withdrawing groups delocalizing the nitrogen lone pair, thereby stabilizing the adduct by reducing the rearomatization driving force further. Indeed, these two derivatives can be purified (if necessary) by column chromatography without any aromatization, whereas the Bn-protected congener aromatizes during attempted chromatographic purification. This latter observation was the driving force behind its isolation by precipitation rather than chromatography in the first instance.

**Figure 6:**
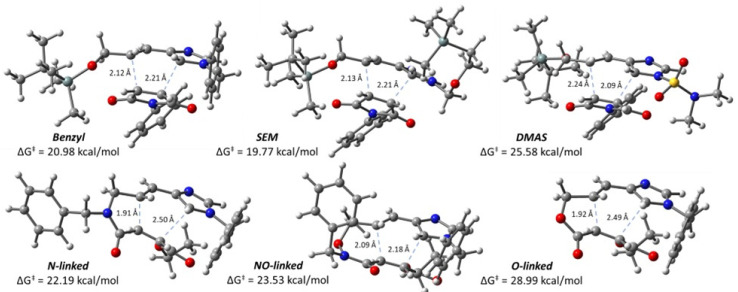
Gas-phase *endo* transition state structures and their corresponding calculated ΔG^‡^ values in kcal/mol.

To compare the relative reactivity of our systems, we undertook an analysis of the energies and structures of the *endo* transition state for each substrate. Comparison of the energies for each protecting group shows a trend of SEM < Bn < DMAS in all solvent systems, which roughly matches the trend observed experimentally, though not all PG/solvent combinations were tested (Figure 6, Supplementary Materials Table S1). The rate differences observed experimentally can likely be attributed to the varying extent of electron donation between the various protecting groups, with Bn being the most donating and DMAS being the least. Inspection of the geometry of the *endo* transition states sheds some light on mechanistic aspects of these cycloadditions with slight divergence between the inter- and intramolecular processes. For the intermolecular reactions, there appears to be slight asynchronicity in the degree of bond formation of the new carbon–carbon bonds, with the polarization of the asynchronicity depending on the protecting group. Both the Bn- and SEM-protected congeners exhibit slightly more advanced bond formation between C5 (diene) and C3 (dienophile), whereas the DMAS-protected derivative is more advanced at C8 (diene) and C4 (dienophile). These data are consistent with the putative polarization of electron density in the diene and the dearomatization barrier.

The calculated transition states of the intramolecular variants also display several interesting features (Figure 6). For example, the N_(amide)_-Bn groups are cis with the carbonyls in the transition states of **10** and **18** so that the dienophile can adopt an appropriate geometry to react. All three transition states exhibit some asynchronicity, but the amide- and ester-linked systems are quite markedly distorted, with the carbon–carbon bond formation on the imidazole end of the diene being distinctly advanced compared to that at the terminal end of the vinyl group. Torsion angles between the internal carbonyl and the electron-deficient C=C indicate that there is a loss of coplanarity in amide **10** (torsion angle = 39.6 °) and ester **14a** (torsion angle = 29.6 °), which is only reflected in an increased barrier for the ester derivative [39,47]. Presumably, this is a consequence of the reduced stabilization of the C=C due to the conjugation of the amide nitrogen to the carbonyl and thereby a reduction in the enthalpic penalty associated with the conformational change. The carbonyl of the ethyl ester remains more or less coplanar with the C=C and thus, in effect, acts as a mono-activated dienophile; previous work from our lab has shown that intermolecular reactions with monosubstituted dienophiles (methyl acrylate and acrylonitrile) are regioselective, placing the activating substituent proximal to the imidazole ring [28]. Moreover, the *N*-substituent on nitrogen increases the reactive rotamer population, providing an additional entropic enhancement [39]. In the hydroxamate substrate, the larger size of the tether results in a formation of a six membered ring, which accommodates the internal carbonyl that remains more or less coplanar with C=C (torsion angle = 0.3 °), which appears to offset the small increase in the entropic barrier for the cyclization. Also, the six membered ring which forms within the hydroxamate tether adopts a boat conformation rather than the initially expected chair-like structure. On closer examination, it can be seen that the Bn group and the carbonyl oxygen are cis, which means that the conformation allows the nitrogen lone pair to overlap with the carbonyl, thereby retaining the amido stabilization.

## 3. Conclusions

In summary, we have established that Diels–Alder reactions of some vinylimidazoles can proceed at room temperature and, if conducted under the right conditions, afford excellent yields of cycloadducts which can be isolated by filtration without undergoing rearomatization. Attempts to extend this observation to intramolecular variants has resulted in mixed outcomes. Amides appear to provide cycloadducts but attempts to obtain the cycloaddition precursors cleanly is compromised by issues with cycloaddition. Mixtures of the initial adduct and the aromatized products are obtained, which, upon attempted purification by chromatography, result in the isolation of only the aromatized adduct. A DFT study of these cycloadditions reveals a higher bias toward *endo* selectivity for the intermolecular cases as compared to the intramolecular cases, which likely explains the low yields obtained for the latter. Examination of the stabilization energy obtained upon rearomatization indicates that this process is very thermodynamically favorable, which, when combined with our ability to isolate the prearomatic cycloadducts for intermolecular derivatives, implies that there is a substantial kinetic barrier for this process. These observations imply that this formal, thermally forbidden [1,3]-H shift likely occurs via a two-step proton transfer pathway. Additionally, DFT calculations show asynchronicity in the transition states with electron donating vs. withdrawing *N*_imid_ protecting groups, showing differences in bond formation at alternate termini in the intermolecular cases and a more pronounced degree of asynchronicity for the intramolecular cases.

## Figures and Tables

**Table 1:** NMR yields for Diels–Alder reactions of vinylimimdazoles **1a**–**c** with NPM as a function of time.

Entry	Compound	PG	Solvent	Time (Days)										
				0	1	2	3	4	5	6	7	8	9	10
				Percent Conversion (%) ^a^										
1	**1a**	Bn ^b^	CDCl_3_	0	20	40	55	66	76	83	87	89	91	89
2	**1b**	DMAS ^b^	CDCl_3_	0	0	0	1	4	7	10	12	12	20	21
3	**1b**		MeOH-d_4_	0	7	14	18	21	23	23	22	20	18	16
4	**1b**		DMSO-d_6_	0	16	31	44	53	63	70	73	76	79	80
5	**1c**	SEM ^c^	C_6_D_6_	0	30	48	59	68	74	76	79	81	81	81
6	**1c**		CDCl_3_	0	17	30	42	48	54	62	68	72	77	79

^a^. Reactions were performed in an NMR tube and the course of the reaction monitored by NMR spectroscopy through integration of appropriate signals (see Supplementary Materials for details). ^b^. Bn- and DMAS-protected Diels–Alder adducts precipitated out of solution when conducted in benzene with isolated yields of 83 and 40%, respectively, after 4 days. ^c^. The cycloadduct was isolated in 70% yield by chromatography.

**Table 2:** Difference between *endo* and *exo* transition state energies in kcal/mol.

Entry	Compound	Protecting Group	*Endo* vs. *Exo* TS Energy Comparison (kcal/mol)				
			Gas Phase	DMSO	Methanol	Chloroform	Benzene
1	**1a**	Bn	4.10	3.91	4.80	4.59	4.33
2	**1b**	DMAS	3.83	3.73	3.48	2.71	2.88
3	**1c**	SEM	5.15	3.54	4.38	4.26	4.30
4	**9**	Bn (amide)	0.77	−0.04	0.30	0.56	0.58
5	**13a**	Bn (ester)	0.96	−0.85	−0.36	−0.59	−0.52
6	**17**	Bn (hydroxamate)	1.24	−0.31	−0.01	0.31	0.56

**Table 3:** Energy of stabilization for the rearomatization process (aromatic adduct—initial prearomatic adduct) in kcal/mol.

Entry	Compound	Protecting Group	Rearomatization Stabilization Energy (kcal/mol)				
			Gas Phase	DMSO	Methanol	Chloroform	Benzene
1	**2a**	Bn	−22.88	−21.68	−20.93	−20.90	−21.34
2	**2b**	DMAS	−17.48	−15.89	−15.28	−15.95	−16.15
3	**2c**	SEM	−22.00	−20.54	−19.09	−19.66	−19.92
4	**10**	Bn (amide)	−24.76	−25.06	−24.84	−25.07	−25.43
5		Bn (ester)	−24.48	−24.76	−24.29	−24.62	−25.07
6	**18**	Bn (hydroxamate)	−24.76	−23.56	−23.43	−23.59	−23.81

## Data Availability

Any reasonable request for original data supporting the work reported in this manuscript will be honored by the corresponding author.

## Supplementary Materials

The following supporting information can be downloaded at: https://www.mdpi.com/article/10.3390/molecules29081902/s1. Stacked ^1^H NMR spectra depicting progress of room temperature DA reactions of **1a**–**c** over time; experimental procedures, characterization data and copies of ^1^H and ^13^C NMR spectra of compounds **1c**, **2a**, **2c**, **11**, **13a**, **14a**, **17**, **19**; details of computational methods and coordinates; details of X-ray crystal structure determination and data listing. Table S1: ΔG^‡^ for endo transition states in kcal/mol. Table S2: ΔG for the formation of prearomatic and aromatic products in kcal/mol. Figure S1: X-ray crystal structure of compound **14a** and atom labelling scheme. Table S3: Crystal data and structure refinement for compound **14a**. Table S4: Bond Lengths for compound **14a**. Table S5: Bond Angles for compound **14a**. The references [28,29,48,49,50,51,52,53,54,55] are cited in Supplementary Materials.
