# Supplementary material for: Room Temperature Diels–Alder Reactions of 4-Vinylimidazoles
Source: Molecules. 2024 Apr 22;29(8):1902. doi: 10.3390/molecules29081902 (PMC11053432; doi:10.3390/molecules29081902)
Supplement: Supplementary file 1 [file molecules-29-01902-s001.zip › molecules-2863419-supplementary (1).pdf]

Room Temperature Diels-Alder Reactions of 4-Vinylimidazoles

Brandon B. Fulton, Alexia J. Hartzell, H.V. Rasika Dias and Carl J. Lovely*

Department of Chemistry and Biochemistry, University of Texas Arlington, Arlington, TX76019, USA

Table of Contents

General Methods 1

Experimental Methods 2

Room Temperature Timed NMR Study Method – General Procedure 2

Room Temperature Timed NMR Spectra........ 2

Synthetic Methods........ 8

Computational Methods........ 14

Additional Tables 15

X-ray Data Collection and Structure Determinations 16

^{1}H and ^{13}C NMR Spectra........ 21

Computational Data........ 35

Cartesian Coordinates for Calculated Gas Phase Endo Transition States........ 35

General Methods

All reagents were purchased from commercial suppliers and were used as received unless otherwise indicated. Anhydrous dichloromethane (DCM) and acetonitrile (MeCN) were obtained by running the previously degassed solvents through a PureSolv solvent system by Inert. Anhydrous tetrahydrofuran (THF) was obtained by drying over sodium through the use of a benzophenone ketyl still. Thin layer chromatography (TLC) was performed on Silica XG aluminum backed plates (thickness: 200 µm) obtained from Sorbtech and 254 nm UV light was used as the visualizing agent. All chromatographic purifications were performed using flash chromatography on silica gel. ^{1}H and ^{13}C nuclear magnetic resonance (NMR) spectra were recorded in $CDCl_3$ (unless otherwise noted) at 500 and 125.8 MHz, respectively (unless otherwise noted) on JEOL instrumentation. Spectra were processed using MestreNova (version 14.1.2) and were referenced utilizing residual undeuterated solvent as the internal reference (i.e. for the case of $CDCl_3$, $CHCl_3$ was used with values of δ = 7.26 for ^{1}H NMR and δ = 77.0 for ^{13}C NMR). Data are reported in terms of chemical shift, multiplicities, and integration. The following abbreviations are used to describe multiplicities: s = singlet, d = doublet, t = triplet, dd = doublet of doublets, ddt = doublet of doublet of triplets, ddd = doublet of doublet of doublets, m = multiplet. Infrared spectra (IR) were recorded neat using a Bruker Alpha series FT-IR spectrometer. High resolution mass spectrometry (HR-MS) was carried out in the Shimadzu Center for Advanced Analytical Chemistry using electrospray ionization for sample introduction and a time-of-flight (TOF) mass analyzer.

Experimental Methods

Room Temperature Timed NMR Study Method – General Procedure

Each protected (Bn, DMAS, and SEM) vinylimidazole[1] (0.06 mmol) was dissolved in deuterated solvent (benzene-d_6, methanol-d_4, DMSO-d_6, and alumina-filtered $CDCl_3$) to achieve a concentration of 0.13 M. *N*-phenylmaleimide (2.5 equiv.) was added and each solution was thoroughly mixed. The solutions were then added to NMR tubes and allowed to stand at room temperature (≈ 25 °C). Spectra were then acquired immediately after addition and subsequently every 24 hours. The relative amounts of unreacted vinylimidazole and cycloadduct were determined by integration of the appropriate signals as indicated by the highlighted protons in their respective stacked spectra.

Room Temperature Timed NMR Spectra

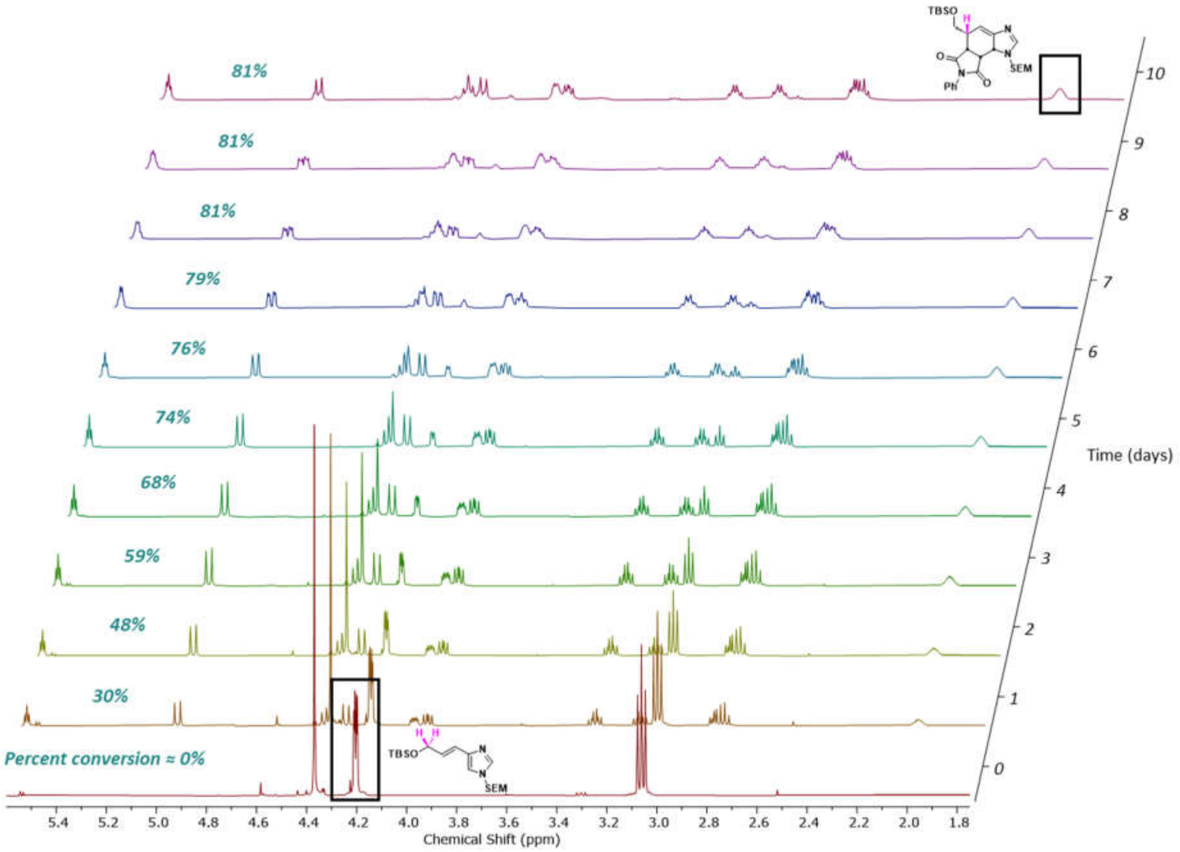

Stacked spectra 1: SEM protected vinylimidazole **1c** in benzene-d_6 with formation of Diels-Alder adduct **2c**. Boxes show the peaks used for integration with the corresponding protons highlighted in pink.

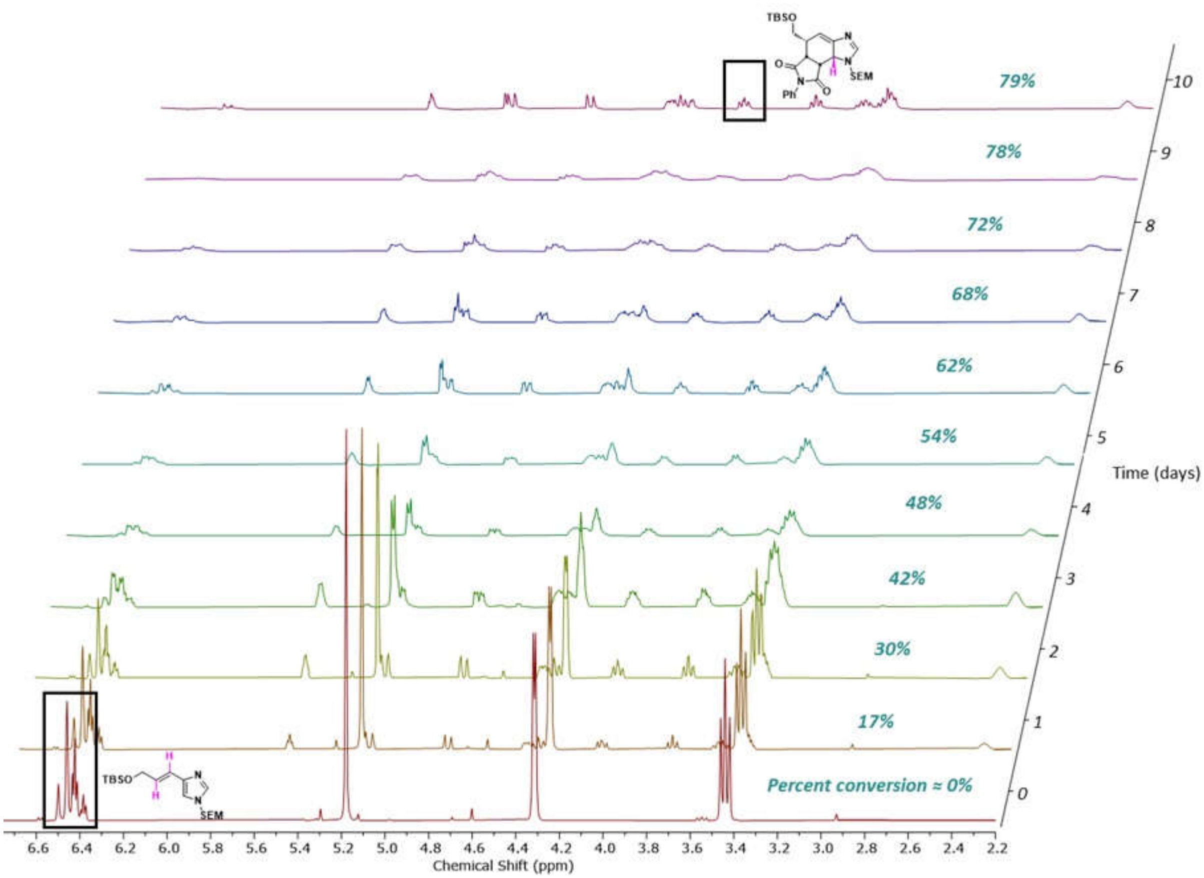

Stacked spectra 2: SEM protected vinylimidazole **1c** in $CDCl_3$ with formation of Diels-Alder adduct **2c**. Boxes show the peaks used for integration with the corresponding protons highlighted in pink.

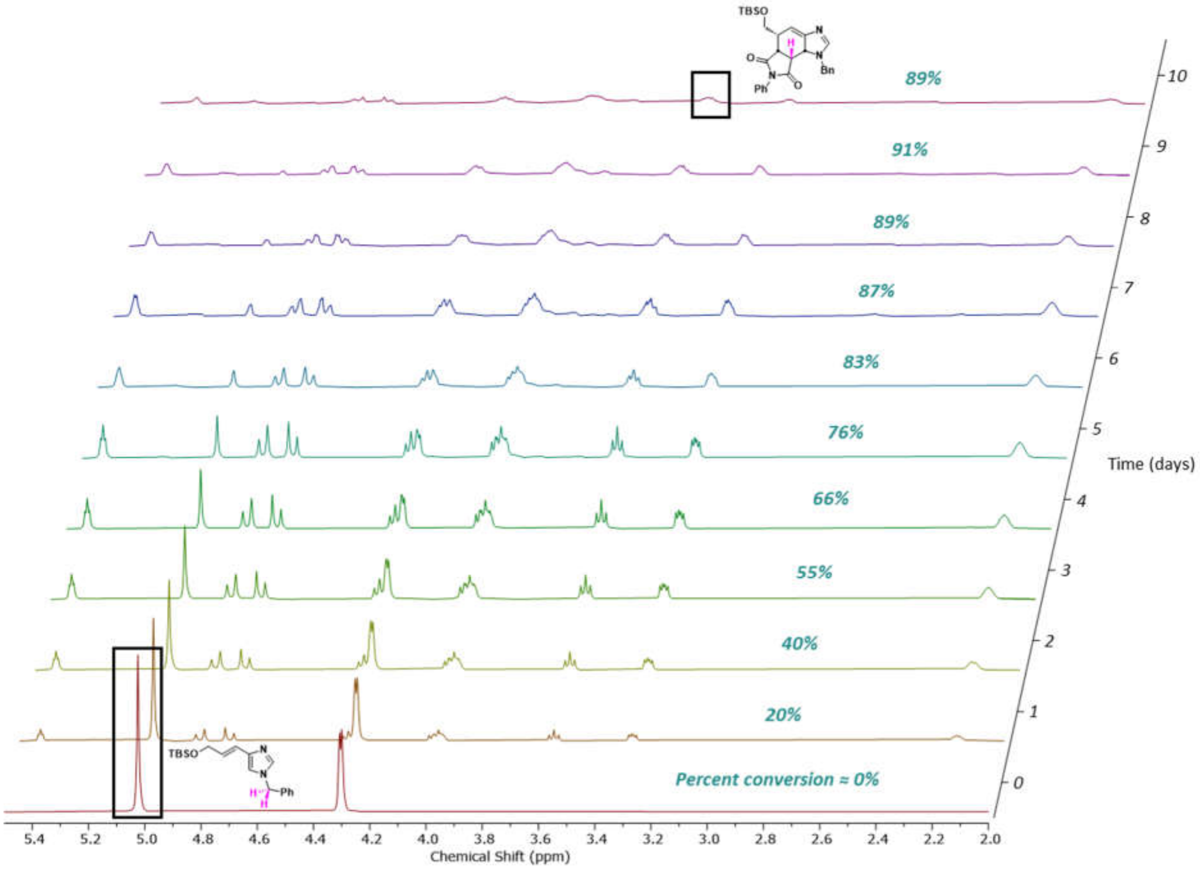

Stacked spectra 3: Bn protected vinylimidazole **1a** in $CDCl_3$ with formation of Diels-Alder adduct **2a**. Boxes show the peaks used for integration with the corresponding protons highlighted in pink.

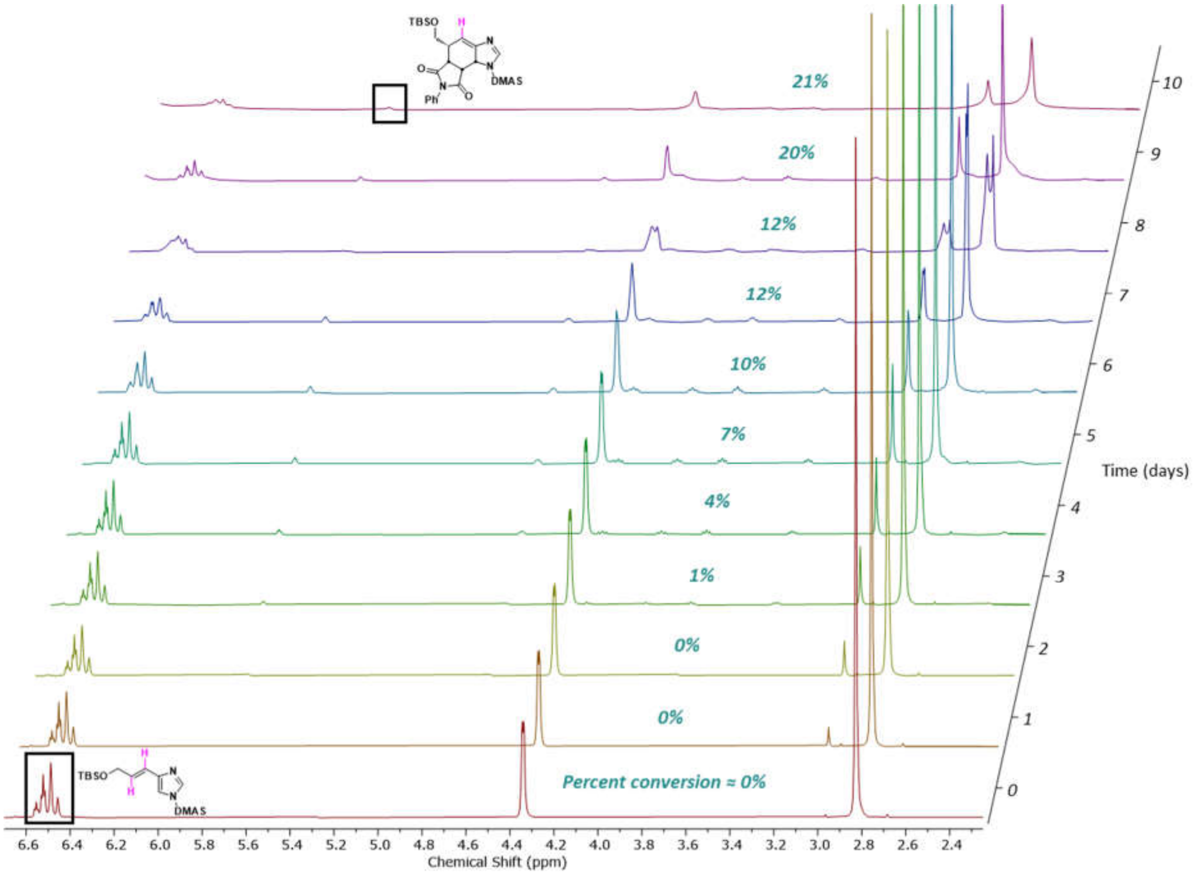

Stacked spectra 4: DMAS protected vinylimidazole **1b** in $CDCl_3$ with formation of Diels-Alder adduct **2b**. Boxes show the peaks used for integration with the corresponding protons highlighted in pink.

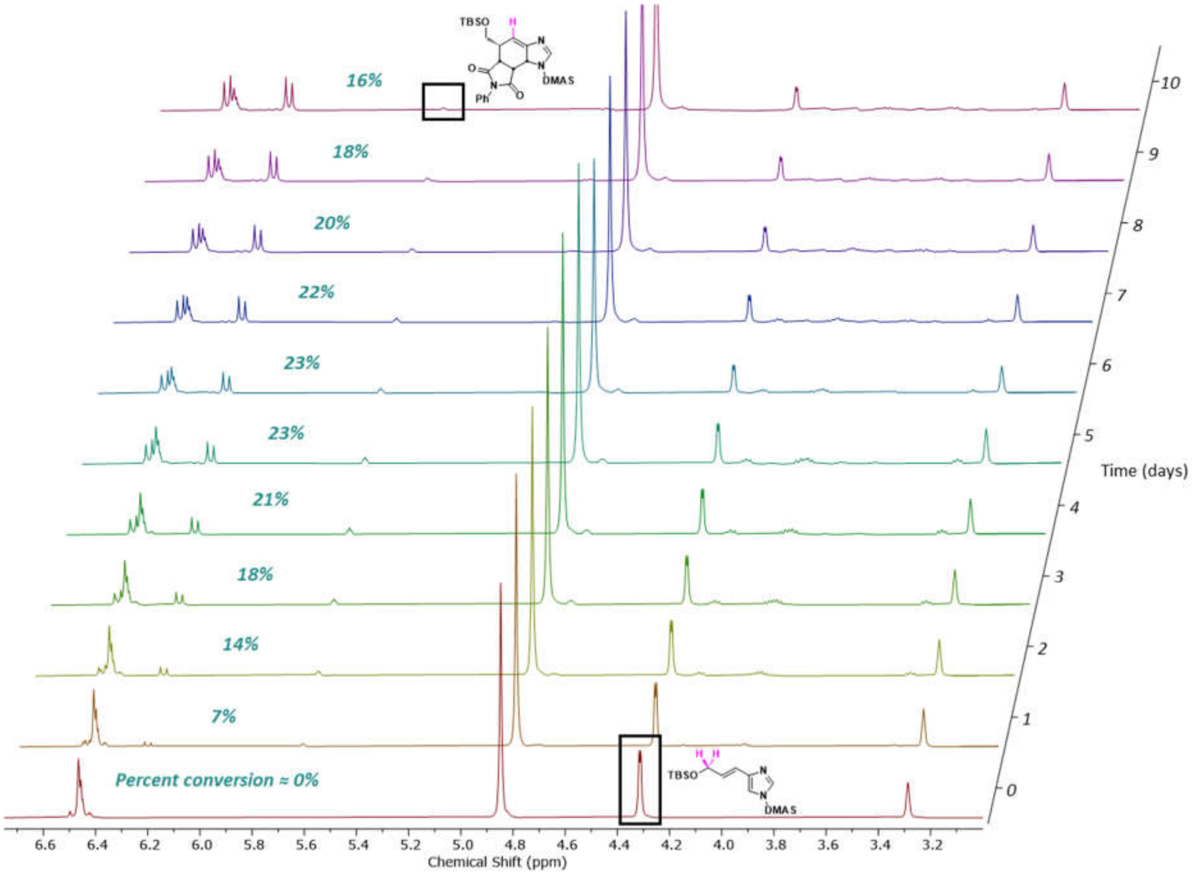

Stacked spectra 5: DMAS protected vinylimidazole **1b** in methanol-d_4 with formation of Diels-Alder adduct **2b**. Boxes show the peaks used for integration with the corresponding protons highlighted in pink.

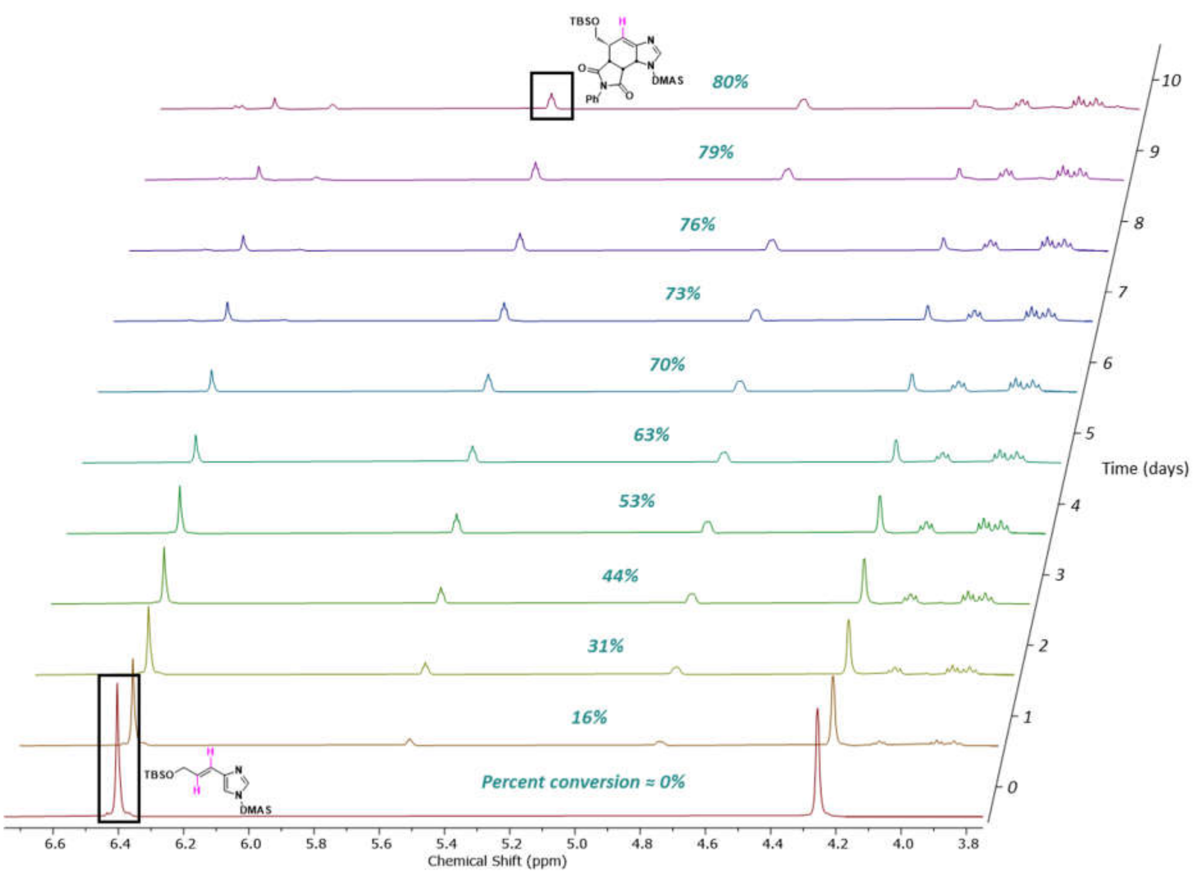

Stacked spectra 6: DMAS protected vinylimidazole **1b** in DMSO-d_6 with formation of Diels-Alder adduct **2b**. Boxes show the peaks used for integration with the corresponding protons highlighted in pink.

Synthetic Methods

*Large Scale Preparation of **2a***

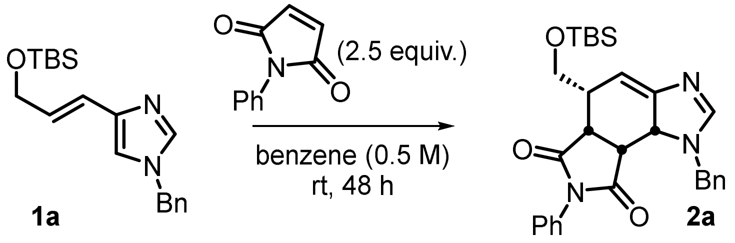

(5R∗,5aS∗,8aS∗,8bS∗)-1-Benzyl-5-tert-butyldimethylsilyloxymethyl-1,5,5a,6,7,8,8a,8b-octahydro-7-phenyl-6,8-dioxopyrrolo[3,4-g]benzimidazole (2a): Vinylimidazole **1a** (21.6 g, 65.8 mmol) and *N*-phenylmaleimide (28.5 g, 164.4 mmol; 2.5 equiv.) were dissolved in benzene (131.5 mL; 0.5 M in **1a**) and stirred at room temperature (≈ 25 °C). After 48 hours, the resulting white precipitate was collected by vacuum filtration and washed with ice-cold benzene to yield the cycloadduct **2a** (30.0 g, 91% yield) as a white solid after being dried under vacuum.[1]

Newly Synthesized Compounds

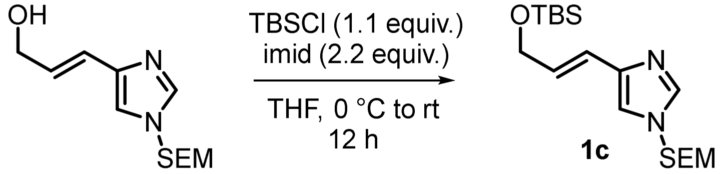

1-(1-(2-(trimethylsilyl)ethoxymethyl))-1H-imidazol-4-yl)-3-tert-butyldimethylsilyloxypropene (1c): The analogous SEM-protected vinylimidazole alcohol[2] (229 mg, 0.90 mmol) was dissolved in anhydrous tetrahydrofuran (5.0 mL) along with imidazole (135 mg, 2.0 mmol; 2.2 equiv.). The solution was cooled to 0 °C and *tert*-butyldimethylsilyl chloride (149 mg, 1.0 mmol; 1.1 equiv.) was added. The reaction mixture was warmed slowly to room temperature (≈ 25 °C) and stirred for an additional 12 hours. The solvent was removed by rotary evaporation and the resulting oil was purified by column chromatography (SiO_2; 3:2 ethyl acetate / hexanes) to yield compound **1c** as a transparent oil (250 mg, 75%).

^{1}H NMR (500 MHz, $CDCl_3$): δ 7.69 (s, 1H), 6.95 (d, *J* = 1.4 Hz, 1H), 6.49 (s, 2H), 5.25 (s, 2H), 4.34 (d, *J* = 1.6 Hz, 2H), 3.48 (t, *J* = 8.2, 7.3 Hz, 2H), 0.92 (s, 9H), 0.89 (m, 2H) 0.09 (s, 6H), -0.02 (s, 9H).

^{13}C NMR (126 MHz, $CDCl_3$): δ 140.8, 137.3, 128.0, 120.8, 115.9, 75.8, 66.2, 63.5, 25.9, 18.3, 17.6, -1.5, -5.3.

FT-IR (neat, cm^{-1}): 2952, 2890, 2856, 1497, 1465, 1359, 1301, 1094, 831, 773.

HRMS (m/z): calculated for $C_{18}H_{36}N_2O_2Si_2$ $[M+H]^+$ 369.2394, found 369.2389.

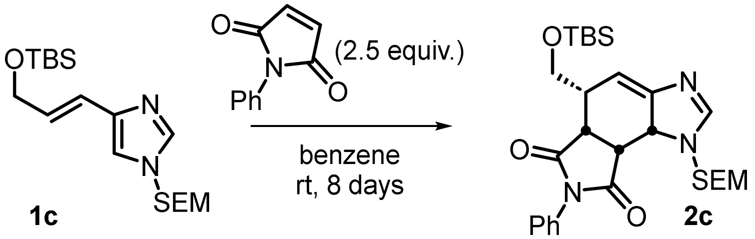

(5R∗,5aS∗,8aS∗,8bS∗)-1-(2-(trimethylsilyl)ethoxymethyl)-5-tert-butyldimethylsilyloxymethyl-1,5,5a,6,7,8,8a,8b-octahydro-7-phenyl-6,8-dioxopyrrolo[3,4-g]benzimidazole (2c): Vinylimidazole **1c** (300 mg, 0.8 mmol) and *N*-phenylmaleimide (352 mg, 20.3 mmol; 2.5 equiv.) were dissolved in benzene (6.3 mL) and stirred at room temperature (≈ 25 °C) for 8 days. The solvent was removed via rotary evaporation and the residue was purified by column chromatography (SiO_2; 7:3 petroleum ether/EtOAc) to yield **2c** (307 mg, 70% yield) as a white solid.

Melting Point: 127-128 °C.

^{1}H NMR (400 MHz, $CDCl_3$): δ 7.44 – 7.37 (m, 2H), 7.35 (d, *J* = 7.0 Hz, 1H), 7.28 – 7.24 (m, 1H), 7.08 (d, *J* = 8.1 Hz, 2H), 5.53 (t, *J* = 4.2 Hz, 1H), 5.18 (d, *J* = 11.6 Hz, 1H), 4.81 (d, *J* = 11.7 Hz, 1H), 4.49 – 4.43 (m, 1H), 4.40 (t, *J* = 8.9 Hz, 1H), 4.10 (dd, *J* = 9.9, 6.9 Hz, 1H), 3.78 (t, *J* = 8.5 Hz, 1H), 3.56 (td, *J* = 9.7, 9.3, 6.2 Hz, 1H), 3.48 – 3.39 (m, 1H), 2.41 – 2.29 (m, 1H), 0.92 (s, 9H), 0.12 (s, 6H), 0.03 (s, 9H).

^{13}C NMR (101 MHz, $CDCl_3$): δ 175.7, 173.7, 159.4, 153.8, 131.6, 129.2, 128.9, 126.7, 106.5, 75.7, 65.9, 62.9, 56.4, 41.7, 41.5, 38.2, 26.1, 18.5, 18.0, -1.3, -5.2, -5.2.

FT-IR (neat, cm^{-1}): 2952, 2857, 1702, 1543, 1396, 1247, 1066, 832.

HRMS (m/z): calculated for $C_{28}H_{43}N_3O_4Si_2$ $[M+H]^+$ 542.2870, found 542.2843.

Method 1:

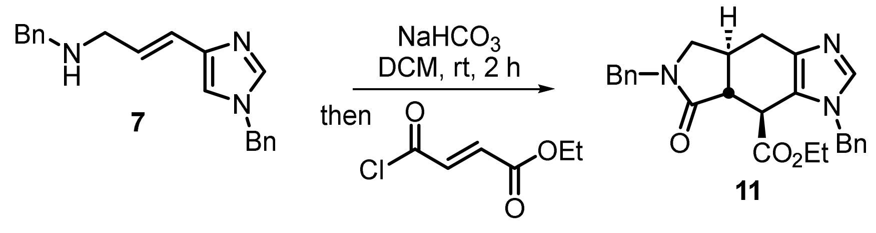

Method 2:

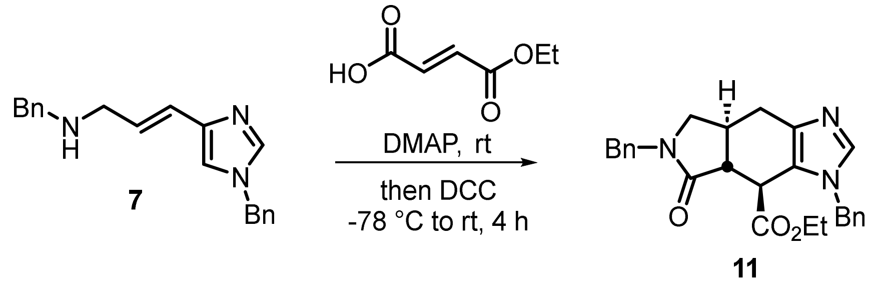

Ethyl (4*S*,4a*R*,7a*S*)-3,6-dibenzyl-5-oxo-3,4,4a,5,6,7,7a,8-octahydroimidazo-[4,5-*f*]isoindole-4-carboxylate (11): *Method 1* (acid chloride): Fumaric acid monoethyl ester **8** (160 mg, 1.1 mmol; 1.1 equiv.) was dissolved in anhydrous dichloromethane (4.6 mL) along with oxalyl chloride (0.5 ml, 5.7 mmol; 5.6 equiv.). Dimethylformamide (3 µL, cat.) was then added and the solution was allowed to stir under an atmosphere of nitrogen at room temperature for 6 hours. After 6 hours, a separate solution containing amine vinylimidazole **7** (320 mg, 1.0 mmol; 1.0 equiv.) and sodium bicarbonate (100 mg) in anhydrous dichloromethane (3 mL) was prepared and stirred at under an atmosphere of nitrogen at room temperature for 2 hours. Once the required time had passed, the solvent of the acid chloride-containing solution was removed by rotary evaporation and remaining oil redissolved in anhydrous diethyl ether (2.3 mL). The vinylimidazole-containing solution was cooled to 0 °C and the ethereal acid chloride solution was added dropwise over a period of 30 minutes. After stirring at 0 °C for 30 minutes, the solution was filtered and the filtrate was concentrated by rotary evaporation. The crude product was then purified by column chromatography (SiO_2; ethyl acetate) to yield a mixture of prearomatic/aromatic Diels-Alder cycloadducts **10** and **11** (168 mg, 37% yield) as an off-white solid. Then sample was then dissolved in $CDCl_3$ and allowed to sit for 3 days. After 5 days, full conversion to aromatized product **11** had occurred.

Method 2 (DCC coupling): Amine vinylimidazole **7** (303 mg, 1.0 mmol; 1.0 equiv.), fumaric acid monoethyl ester **8** (216 mg, 1.5 mmol; 1.5 equiv.), and 4-dimethylaminopyridine (5 mg, 0.04 mmol; 0.04 equiv.) were dissolved in anhydrous dichloromethane (1.5 mL) and kept under an atmosphere of nitrogen. The solution was cooled to -78 °C and a solution of *N*,*N'*-dicyclohexylcarbodiimide (250 mg, 1.2 mmol; 1.2 equiv.) in anhydrous dichloromethane (0.75 mL) was added. The solution was slowly warmed to room temperature and allowed to stir for a total of 4 hours. The cloudy solution was then filtered through a pad of Celite and the filtrate was concentrated by rotary evaporation. A ^1H NMR spectrum of the crude product revealed what appeared to be the unreacted Diels-Alder precursor. However, after purifying the crude product by column chromatography (SiO_2; ethyl acetate), aromatized Diels-Alder cycloadduct **11** (182 mg, 42% yield; off-white solid) was isolated as the sole product.

Melting Point: 184-185 °C

^{1}H NMR (500 MHz, $CDCl_3$): δ 7.48 (s, 1H), 7.36 – 7.24 (m, 6H), 7.20 (d, *J* = 7.4 Hz, 2H), 7.04 (d, *J* = 7.6 Hz, 2H), 5.11 (d, *J* = 15.9 Hz, 1H), 4.96 (d, *J* = 15.9 Hz, 1H), 4.49 (d, *J* = 14.7 Hz, 1H), 4.38 (d, *J* = 14.7 Hz, 1H),

4.15 – 4.00 (m, 2H), 3.70 (d, *J* = 10.9 Hz, 1H), 3.36 – 3.29 (m, 1H), 3.11 (t, *J* = 9.8 Hz, 1H), 2.96 (t, *J* = 12.0 Hz, 1H), 2.84 (d, *J* = 10.5 Hz, 1H), 2.63 (t, *J* = 13.1 Hz, 1H), 2.24 (m, 1H), 2.05 – 2.00 (m, 1H), 1.26 (t, *J* = 7.4 Hz, 3H).

^{13}C NMR (126 MHz, $CDCl_3$): δ 172.6, 171.9, 138.3, 136.2, 135.5, 129.0, 128.8, 128.3, 128.2, 127.8, 126.8, 123.6, 61.9, 49.8, 49.2, 49.1, 46.7, 41.2, 37.5, 28.0, 14.1.

FT-IR (neat, cm^{-1}): 3323, 2926, 2849, 1623, 1567, 1437, 1309, 1242, 1086, 891, 640.

HRMS (m/z): calculated for $C_{26}H_{27}N_3O_3$ $[M+H]^+$ 430.2131, found 430.2117.

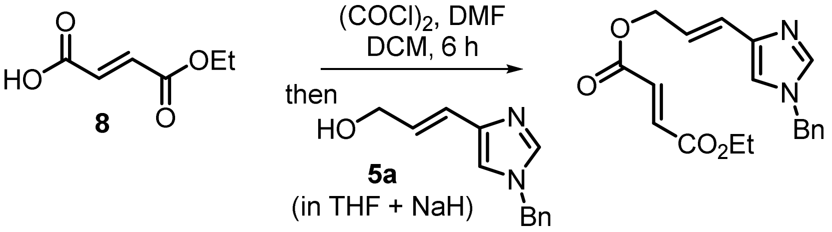

Ethyl (2*E*)-3-(1-benzyl-1H-imidazol-4-yl)-2-propenyl (2*E*)-2-butenedioate (13a): Fumaric acid monoethyl ester **8** (570 mg, 4.0 mmol; 1.05 equiv.) was dissolved in anhydrous dichloromethane (14.4 mL) along with oxalyl chloride (1.5 mL, 17.5 mmol; 4.4 equiv. to ester). Dimethylformamide (9.4 µL, cat.) was then added and the solution was allowed to stir under an atmosphere of nitrogen at room temperature for 6 hours. After 6 hours, alcohol vinylimidazole **5a** (808 mg, 3.8 mmol; 1.0 equiv.) was dissolved in anhydrous tetrahydrofuran (33 mL) and cooled to 0 °C under an atmosphere of nitrogen. After 30 minutes at 0 °C, sodium hydride (60% dispersion in mineral oil; 227 mg, 5.7 mmol; 1.5 equiv.) was added portion wise. This solution was allowed to stir for 1 hour at 0 °C and then the acid chloride solution was added dropwise at this same temperature. The solvent was then removed by rotary evaporation and the crude product was purified by column chromatography (SiO_2; 1:1 ethyl acetate/hexanes) to yield compound **13a** (542 mg, 42% yield) as an off-white solid.

Melting Point: 96-97 °C.

^{1}H NMR (500 MHz, $CDCl_3$): δ 7.58 (s, 1H), 7.34 (d, *J* = 7.4 Hz, 3H), 7.15 (d, *J* = 6.7 Hz, 2H), 6.85 (s, 3H), 6.54 (d, *J* = 15.8 Hz, 1H), 6.47 – 6.37 (m, 1H), 5.07 (s, 2H), 4.80 (d, *J* = 6.4 Hz, 2H), 4.24 (q, *J* = 7.1 Hz, 2H), 1.30 (t, *J* = 7.2 Hz, 3H).

^{13}C NMR (126 MHz, $CDCl_3$): δ 165.1, 164.9, 139.5, 137.8, 135.8, 134.0, 133.6, 129.2, 128.6, 127.5, 126.1, 121.3, 117.8, 65.9, 61.4, 51.2, 14.2.

FT-IR (neat, cm^{-1}): 3105, 3069, 2982, 2926, 1704, 1640,1449, 1285, 1262, 1171,995, 737.

HRMS (m/z): calculated for $C_{19}H_{21}N_2O_4$ $[M+H]^+$ 341.1501, found 341.1485.

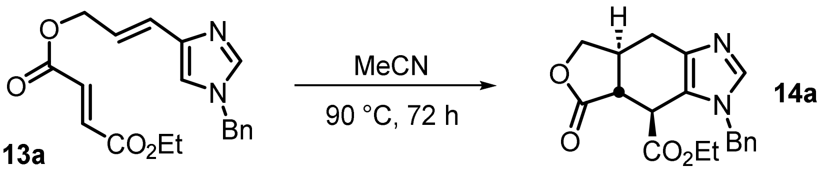

Ethyl (4a*S,7a*R**,8*S**)-1-benzyl-7-oxo-4,4a,7a,8-tetrahydro-1*H*-furo[3,4-*f*]benz-imidazole-8-carboxylate (14a):** Ester-linked Diels-Alder precursor **13a** (200 mg, 0.6 mmol) was dissolved in acetonitrile (0.5 mL) and stirred at 90 °C for 72 hours. After cooling to room temperature, column chromatography (SiO_2; 1:1 ethyl acetate/hexanes to 8:2 ethyl acetate/methanol) was utilized to yield **14a** (120 mg, 60%) as a white solid.

Melting Point: 123-124 °C.

^{1}H NMR (500 MHz, $CDCl_3$): δ 7.44 (s, 1H), 7.37 – 7.23 (m, 3H), 6.99 (d, *J* = 6.7 Hz, 1H), 5.10 (d, *J* = 16.0 Hz, 1H), 4.97 (d, *J* = 16.2 Hz, 1H), 4.50 (dd, *J* = 8.7, 6.9 Hz, 1H), 4.06 (dd, *J* = 11.0, 8.8 Hz, 1H), 4.02 – 3.92 (m, 2H), 3.67 (d, *J* = 10.7 Hz, 1H), 3.04 (dd, *J* = 13.7, 10.7 Hz, 1H), 2.90 (dd, *J* = 14.7, 4.9 Hz, 1H), 2.68 (t, *J* = 14.4 Hz, 1H), 2.61 – 2.48 (m, 1H), 1.19 (t, *J* = 7.1 Hz, 3H).

^{13}C NMR (126 MHz, $CDCl_3$): δ 174.5, 170.8, 138.8, 138.4, 135.5, 129.0, 128.2, 126.6, 122.8, 71.4, 62.1, 49.0, 46.3, 40.3, 39.5, 27.2, 13.9.

FT-IR (neat, cm^{-1}): 3350, 2983, 2907, 1175, 1127, 1495, 1448, 1368, 1303, 1258, 1167, 1083, 1024, 990, 733, 702.

HRMS (m/z): calculated for $C_{19}H_{21}N_2O_4$ $[M+H]^+$ 341.1501, found 341.1489.

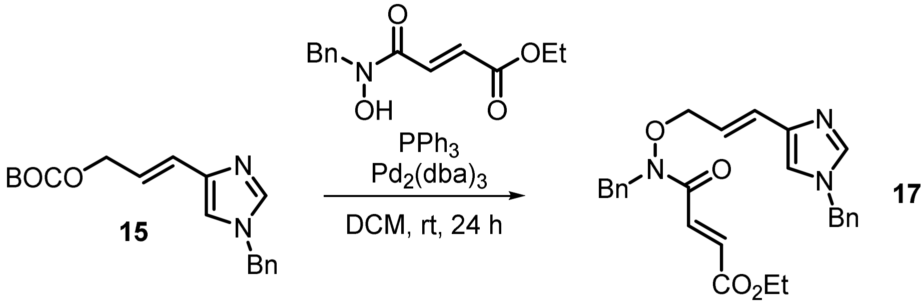

Ethyl (*E*)-4-(benzyl(((*E*)-3-(1-benzyl-1*H*-imidazol-4-yl)allyl)oxy)amino)-4-oxobut-2-enoate (17): Boc-protected vinylimidazole **16** (500 mg, 1.6 mmol), half-hydroxamic acid **15**[3] (396 mg, 1.6 mmol; 1.0 equiv.), and triphenylphosphine (56 mg, 0.2 mmol; 0.13 equiv.) were dissolved in anhydrous dichloromethane (28 mL). The reaction mixture was degassed by bubbling nitrogen gas through the solution. tris(dibenzylideneacetone)dipalladium(0) (44 mg, 0.05 mmol; 0.03 equiv.) was then added and the reaction was allowed to stir for 24 hours at room temperature. The solution was then filtered through a pad of Celite and the filtrate was concentrated by rotary evaporation. The crude product was purified by column chromatography (SiO_2; gradient 1:1 to 2:1 ethyl acetate/hexanes) to yield compound **17** (638 mg, 90% yield) as an orange oil.

^{1}H NMR (500 MHz, $CDCl_3$): δ 7.54 (s, 1H), 7.51 (d, *J* = 14.9 Hz, 1H), 7.39 – 7.25 (m, 8H), 7.15 (d, *J* = 7.7 Hz, 2H), 6.89 (d, *J* = 15.6 Hz, 1H), 6.84 (d, *J* = 1.4 Hz, 1H), 6.44 (d, *J* = 15.8 Hz, 1H), 6.41 – 6.31 (m, 1H), 5.07 (s, 2H), 4.88 (s, 2H), 4.40 (d, *J* = 6.6 Hz, 2H), 4.22 (q, *J* = 7.1 Hz, 2H), 1.28 (t, *J* = 7.1 Hz, 3H).

^{13}C NMR (126 MHz, $CDCl_3$): δ 165.6, 165.4, 139.3, 137.9, 136.0, 135.7, 132.6, 132.3, 129.2, 128.7, 128.6, 128.4, 127.9, 127.4, 120.0, 118.2, 76.8, 61.2, 51.1, 50.6, 14.22.

FT-IR (neat, cm^{-1}): 3062, 3031, 2980, 2931, 2871, 1719, 1656, 1543, 1299, 1164, 969 698.

HRMS (m/z): calculated for $C_{26}H_{28}N_3O_4$ $[M+H]^+$ 446.2080, found 446.2060.

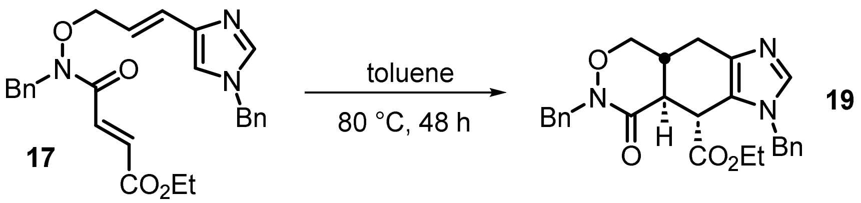

Ethyl (4a*R*,8a*S*,9*R*)-1,7-dibenzyl-8-oxo-1,4,4a,5,7,8,8a,9-octahydroimidazo-[4',5':4,5]benzo[1,2-*d*][1,2]oxazine-9-carboxylate (19): Hydroxamate-linked Diels-Alder precursor **17** (58 mg, 0.1 mmol) was dissolved in toluene (1 mL) and stirred at 80 °C for 48 hours. The reaction mixture was purified by column chromatography (SiO_2; gradient ethyl acetate to 9:1 ethyl acetate/methanol) to yield **19** (17 mg, 30% yield) as a red oil which was contaminated with traces of what is likely the *exo* Diels-Alder adduct.

^{1}H NMR (500 MHz, $CDCl_3$): δ 7.39 (s, 1H), 7.36 – 7.27 (m, 8H), 7.02 (d, *J* = 7.3 Hz, 2H), 5.20 (d, *J* = 16.3 Hz, 1H), 5.13 (d, *J* = 16.2 Hz, 1H), 4.83 (d, *J* = 15.2 Hz, 1H), 4.67 (d, *J* = 15.2 Hz, 1H), 4.11 – 3.92 (m, 4H), 3.92 – 3.82 (m, 2H), 3.38 (dd, *J* = 12.0, 10.2 Hz, 1H), 2.75 (d, *J* = 19.8 Hz, 1H), 2.23 (s, 1H), 1.19 (t, *J* = 7.2 Hz, 3H).

^{13}C NMR (126 MHz, $CDCl_3$): δ 171.9, 170.5, 136.2, 135.7, 129.0, 129.0, 128.7, 128.6, 128.5, 128.1, 128.0, 127.4, 126.5, 74.8, 49.8, 48.9, 44.6, 40.7, 36.9, 31.7, 30.5, 14.0.

FT-IR (neat, cm^{-1}): 3033, 2977, 2929, 1729, 1669, 1495, 1443, 1241, 1182, 1023, 729, 699.

HRMS (m/z): calculated for $C_{26}H_{28}N_3O_4$ $[M+H]^+$ 446.2080, found 446.2066.

Computational Methods

Input structures were created with the molecular modelling program Avogadro.[4] These input structures were then subjected to xTB's CREST module (eXtended Tight Binding / Conformer Ensemble Sampling Tool) using the default settings (energy window = 6.0 kcal/mol, energy threshold = 0.05 kcal/mol, RMSD threshold = 0.125 Å) to generate a set of low energy conformers.[5, 6] The lowest energy conformation was used as a starting point for geometry optimization and frequency calculations which were carried out with Gaussian 16 at the M06-2X/6-31G(d) level of theory in the gas phase for all starting materials, initial cycloadducts, and rearomatized products.[7] Transition state structures were located using Gaussian's QST3 method at the same level of theory and the identity of these structures as saddle points was verified by the observation of a single negative vibrational frequency corresponding to the mode of vibration characteristic of [4+2] cycloaddition reactions. Solvation energies were then computed at the same level of theory by incorporating the Solvation Model based on Density (SMD) for each solvent. Higher level single point energies and thermal corrections to Gibbs free energies were computed at the M06-2X/6-311+G(d,p) level of theory. Finally, energies were calculated using equations (1) and (2).

(1) $$E = E_{SP} + \Delta G + \Delta E_{SMD}$$

(2) $$\Delta E_{SMD} = E_{solvent} - E_{gas-phase}$$

Additional Tables

Table S1: ΔG‡ for endo transition states in kcal/mol.

Protecting Group	ΔG‡ TS energies (kcal/mol)									
	Gas Phase		DMSO		Methanol		Chloroform		Benzene	
	endo	exo	endo	exo	endo	exo	endo	exo	endo	exo
**Bn**	20.98	25.08	26.99	30.90	24.12	28.92	26.12	30.72	26.14	30.47
**SEM**	19.77	24.92	25.61	29.15	23.35	27.73	24.38	28.64	24.12	28.42
**DMAS**	25.58	29.41	30.83	34.56	28.17	31.65	30.98	33.69	30.96	33.84
**N-linked**	22.19	22.96	25.07	25.02	21.88	22.17	23.42	23.98	23.75	24.33
**NO-linked**	23.53	24.77	22.69	22.38	21.66	21.65	22.93	23.24	23.42	23.98
**O-linked**	28.99	29.95	30.26	29.41	27.91	27.56	31.02	30.44	31.63	31.11

Table S2: ΔG for the formation of prearomatic and aromatic products in kcal/mol.

Protecting Group		ΔG (kcal/mol)				
		Gas Phase	DMSO	Methanol	Chloroform	Benzene
**Bn**	prearomatic	-12.41	-6.90	-9.43	-7.82	-7.79
	aromatic	-35.28	-28.58	-30.35	-28.72	-29.13
**SEM**	prearomatic	-14.46	-9.71	-12.63	-10.71	-10.70
	aromatic	-36.45	-30.25	-31.72	-30.37	-30.62
**DMAS**	prearomatic	-14.27	-9.90	-13.00	-9.84	-9.57
	aromatic	-31.74	-25.78	-28.28	-25.80	-25.73
**N-linked**	prearomatic	-11.16	-9.02	-11.38	-10.22	-9.79
	aromatic	-35.92	-34.08	-36.22	-35.28	-35.22
**NO-linked**	prearomatic	-11.16	-10.13	-10.58	-9.16	-8.40
	aromatic	-35.92	-33.70	-34.01	-32.76	-32.21
**O-linked**	prearomatic	-4.48	-4.45	-5.89	-3.08	-2.28
	aromatic	-28.96	-29.20	-30.17	-27.70	-27.35

X-ray Data Collection and Structure Determinations

A suitable crystal of compound **14a** covered with a layer of hydrocarbon/Paratone-N oil was selected and mounted on a Cryo-loop, and immediately placed in the low temperature nitrogen stream. The X-ray intensity data were measured at 100(2) K (unless otherwise noted) Bruker D8 Quest equipped with a PHOTON II 7 CPAD detector and an Oxford Cryosystems 700 series cooler, a Triumph monochromator, and a Mo Ka fine-focus sealed tube (λ = 0.71073 Å). Intensity data were processed using the Bruker Apex program suite. Absorption corrections were applied by using SADABS.[8] Initial atomic positions were located by SHELXT,[9] and the structures of the compounds were refined by the least-squares method using SHELXL[10] within Olex2 GUI.[11] All the non-hydrogen atoms were refined anisotropically. Hydrogen atoms were included at calculated positions and refined using appropriate riding models. X-ray structural figures were generated using Olex2.[11] The CCDC 2327107 contain the supplementary crystallographic data for these molecules. Selected structural data and data collection information for **14a** are also included in the Supporting Information.

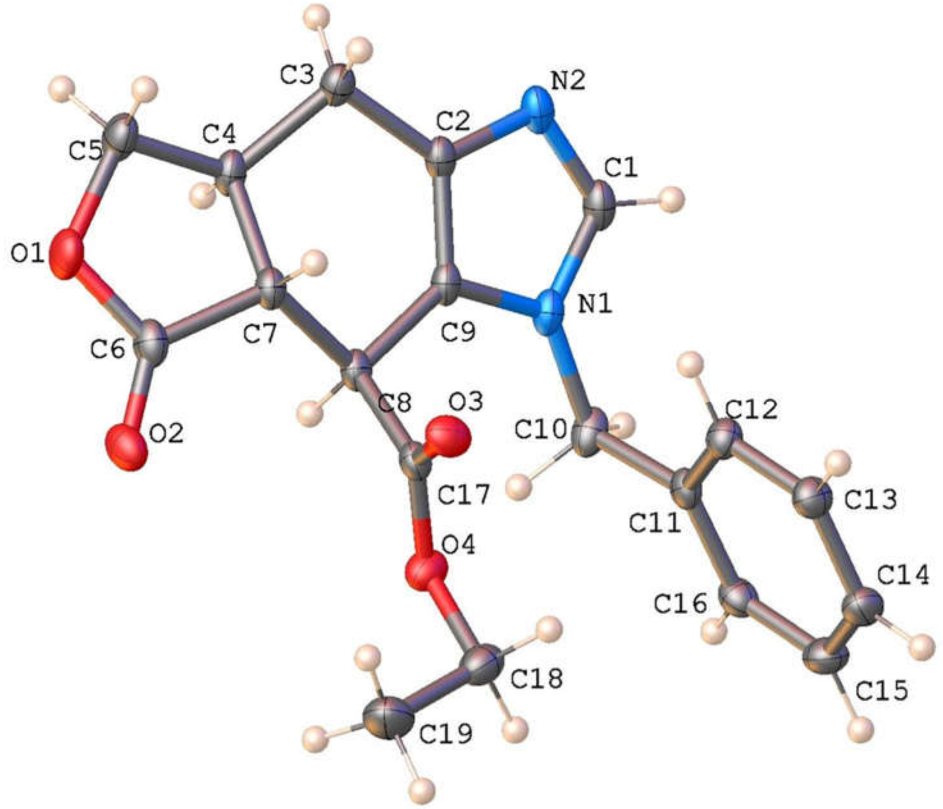

Figure S1: X-ray crystal structure of compound **14a** and atom labelling scheme.

Table S3: Crystal data and structure refinement for compound **14a**.

Identification code	HRD148_0m_a
Empirical formula	$C_{19}H_{20}N_2O_4$
Formula weight	340.37
Temperature/K	100.00
Crystal system	monoclinic
Space group	$P2_1/c$
a/Å	8.5839(3)
b/Å	20.1591(6)
c/Å	9.5816(3)
α/°	90
β/°	92.781(2)
γ/°	90
Volume/Å^3	1656.08(9)
Z	4
ρ_{calc}g/cm^3	1.365
μ/mm^{-1}	0.097
F(000)	720.0
Crystal size/mm^3	0.27 × 0.2 × 0.12
Radiation	Mo Kα (λ = 0.71073)
2Θ range for data collection/°	5.87 to 61.098
Index ranges	-12 ≤ h ≤ 12, -28 ≤ k ≤ 28, -13 ≤ l ≤ 13
Reflections collected	27917
Independent reflections	5056 [R_{int} = 0.0230, R_{sigma} = 0.0161]
Data/restraints/parameters	5056/0/227
Goodness-of-fit on F^2	1.077
Final R indexes [I>=2σ (I)]	R_1 = 0.0400, wR_2 = 0.1082
Final R indexes [all data]	R_1 = 0.0446, wR_2 = 0.1119
Largest diff. peak/hole / e Å^{-3}	0.46/-0.23

Table S4: Bond Lengths for compound **14a**.

Atom	Atom	Length/Å	Atom	Atom	Length/Å
O1	C5	1.4663(14)	C4	C5	1.5233(14)
O1	C6	1.3501(13)	C4	C7	1.5297(14)
O2	C6	1.2055(14)	C6	C7	1.5058(13)
O3	C17	1.2093(12)	C7	C8	1.5269(13)
O4	C17	1.3301(12)	C8	C9	1.5077(13)
O4	C18	1.4639(13)	C8	C17	1.5286(13)
N1	C1	1.3580(14)	C10	C11	1.5097(14)
N1	C9	1.3866(12)	C11	C12	1.3918(13)
N1	C10	1.4568(13)	C11	C16	1.3945(14)
N2	C1	1.3185(14)	C12	C13	1.3945(14)
N2	C2	1.3827(12)	C13	C14	1.3897(14)
C2	C3	1.4986(13)	C14	C15	1.3930(15)
C2	C9	1.3737(14)	C15	C16	1.3854(15)
C3	C4	1.5253(14)	C18	C19	1.4991(18)

Table S5: Bond Angles for compound **14a**.

Atom	Atom	Atom	Angle/°	Atom	Atom	Atom	Angle/°
C6	O1	C5	109.97(8)	C8	C7	C4	114.42(8)
C17	O4	C18	115.87(8)	C7	C8	C17	108.73(7)
C1	N1	C9	106.40(8)	C9	C8	C7	104.40(8)
C1	N1	C10	125.25(9)	C9	C8	C17	113.29(7)
C9	N1	C10	128.14(9)	N1	C9	C8	127.44(9)
C1	N2	C2	104.70(9)	C2	C9	N1	105.57(8)
N2	C1	N1	112.85(9)	C2	C9	C8	126.94(8)
N2	C2	C3	124.15(9)	N1	C10	C11	115.08(8)
C9	C2	N2	110.47(9)	C12	C11	C10	123.49(9)
C9	C2	C3	125.07(9)	C12	C11	C16	119.31(9)
C2	C3	C4	105.70(8)	C16	C11	C10	117.20(9)
C3	C4	C7	110.39(8)	C11	C12	C13	120.11(9)
C5	C4	C3	119.92(9)	C14	C13	C12	120.43(9)
C5	C4	C7	100.29(8)	C13	C14	C15	119.31(10)
O1	C5	C4	104.20(8)	C16	C15	C14	120.38(10)
O1	C6	C7	109.05(9)	C15	C16	C11	120.45(9)
O2	C6	O1	121.85(9)	O3	C17	O4	124.57(9)
O2	C6	C7	129.09(10)	O3	C17	C8	123.84(9)
C6	C7	C4	102.25(8)	O4	C17	C8	111.58(8)
C6	C7	C8	116.45(8)	O4	C18	C19	110.52(9)

References

(1) Lovely, C. J.; Du, H.; Sivappa, R.; Bhandari, M. R.; He, Y.; Dias, H. V. R. Preparation and Diels−Alder Chemistry of 4-Vinylimidazoles. *J. Org. Chem.* **2007**, *72*, 3741-3749.
(2) Sivappa, R.; Hernandez, N. M.; Yong He; Lovely, C. J. Studies toward the Total Synthesis of Axinellamine and Massadine. *Org. Lett.* **2007**, *9*, 3861-3864.
(3) Clarke, P. A.; Cridland, A. P.; Rolla, G. A.; Iqbal, M.; Bainbridge, N. P.; Whitwood, A. C.; Wilson, C. Studies on the Synthesis of the Abc Rings of (±)-Hexacyclinic Acid. *J. Org. Chem.* **2009**, *74*, 7812-7821.
(4) Hanwell, M. D.; Curtis, D. E.; Lonie, D. C.; Vandermeersch, T.; Zurek, E.; Hutchison, G. R. Avogadro: An Advanced Semantic Chemical Editor, Visualization, and Analysis Platform. *J. Cheminform* **2012**, *4*, 1-17.
(5) Grimme, S. Exploration of Chemical Compound, Conformer, and Reaction Space with Meta-Dynamics Simulations Based on Tight-Binding Quantum Chemical Calculations. *J. Chem. Theory Comput.* **2019**, *15*, 2847-2862.
(6) Pracht, P.; Bohle, F.; Grimme, S. Automated Exploration of the Low-Energy Chemical Space with Fast Quantum Chemical Methods. *Phys. Chem. Chem. Phys.* **2020**, *22*, 7169-7192.
(7) Frisch, M. J., et al. *Gaussian 16, Revision C.01*. Gaussian, Inc., Wallingford CT, 2016., 2016. https://gaussian.com/citation/ (accessed October 23, 2023).
(8) Krause, L.; Herbst-Irmer, R.; Sheldrick, G. M.; Stalke, D. Comparison of Silver and Molybdenum Microfocus X-Ray Sources for Single-Crystal Structure Determination. *J. Appl. Crystallogr.* **2015**, *48*, 3-10.
(9) Sheldrick, G. M. Shelxt–Integrated Space-Group and Crystal-Structure Determination. *Acta Cryst. A* **2015**, *71*, 3-8.
(10) Sheldrick, G. M. Crystal Structure Refinement with Shelxl. *Acta Cryst. C* **2015**, *71*, 3-8.
(11) Dolomanov, O. V.; Bourhis, L. J.; Gildea, R. J.; Howard, J. A.; Puschmann, H. Olex2: A Complete Structure Solution, Refinement and Analysis Program. *J. Appl. Crystallogr.* **2009**, *42*, 339-341.

^{1}H and ^{13}C NMR Spectra

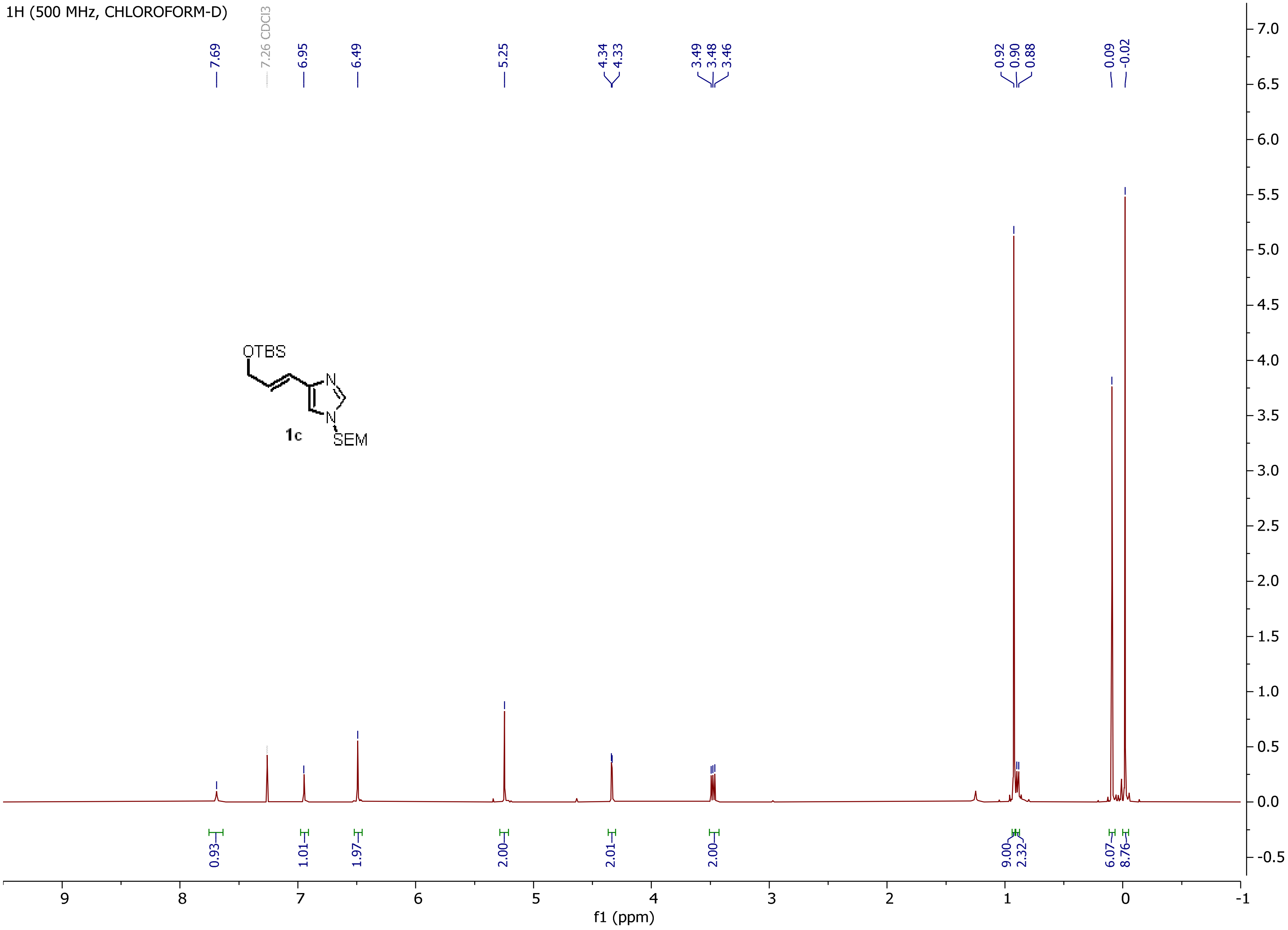

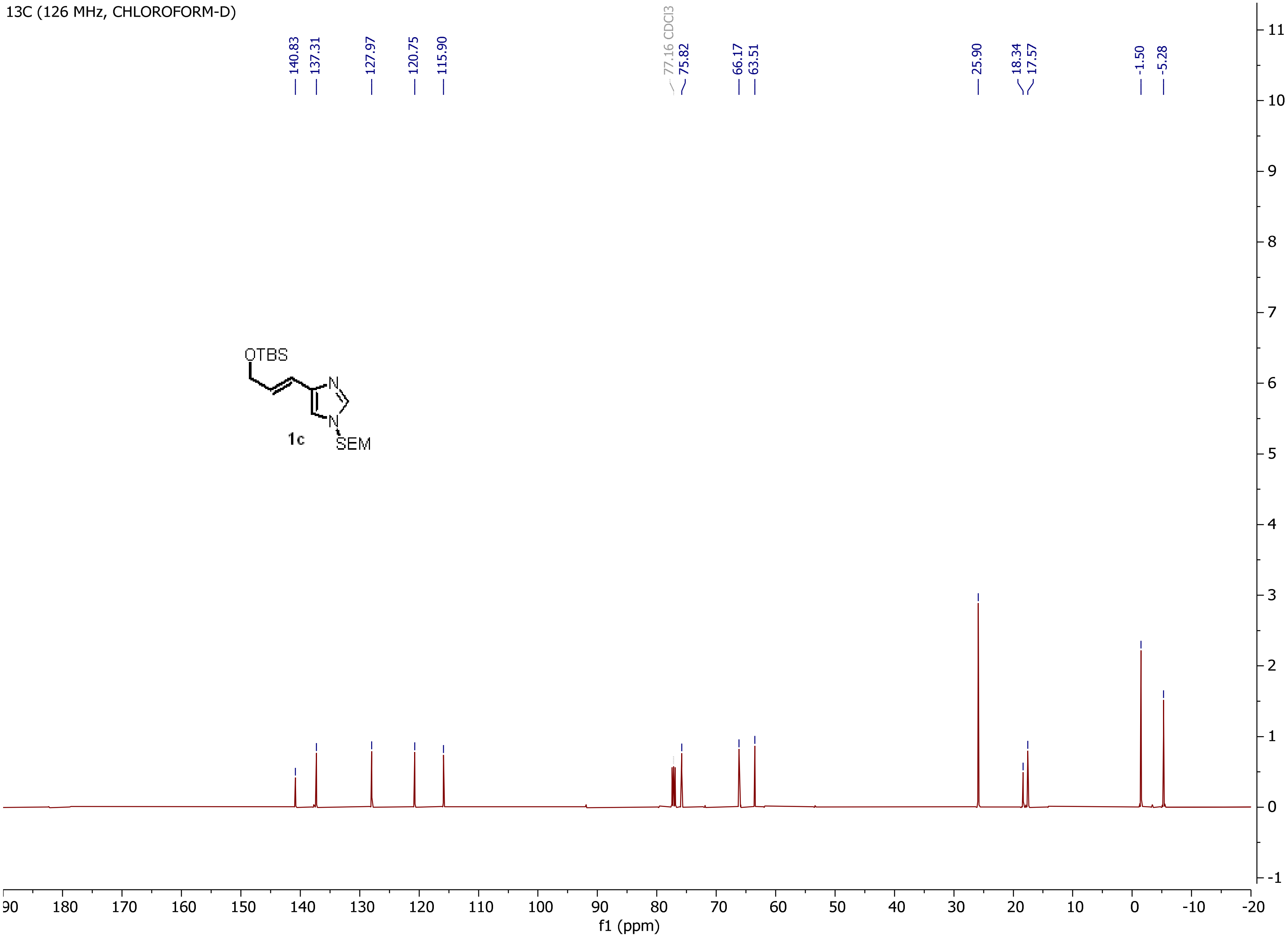
13C (126 MHz, CHLOROFORM-D)
140.83
137.31
127.97
120.75
115.90
77.16 CDCl3
75.82
66.17
63.51
25.90
18.34
17.57
-1.50
-5.28
OTBS
N
N
SEM
1c
f1 (ppm)

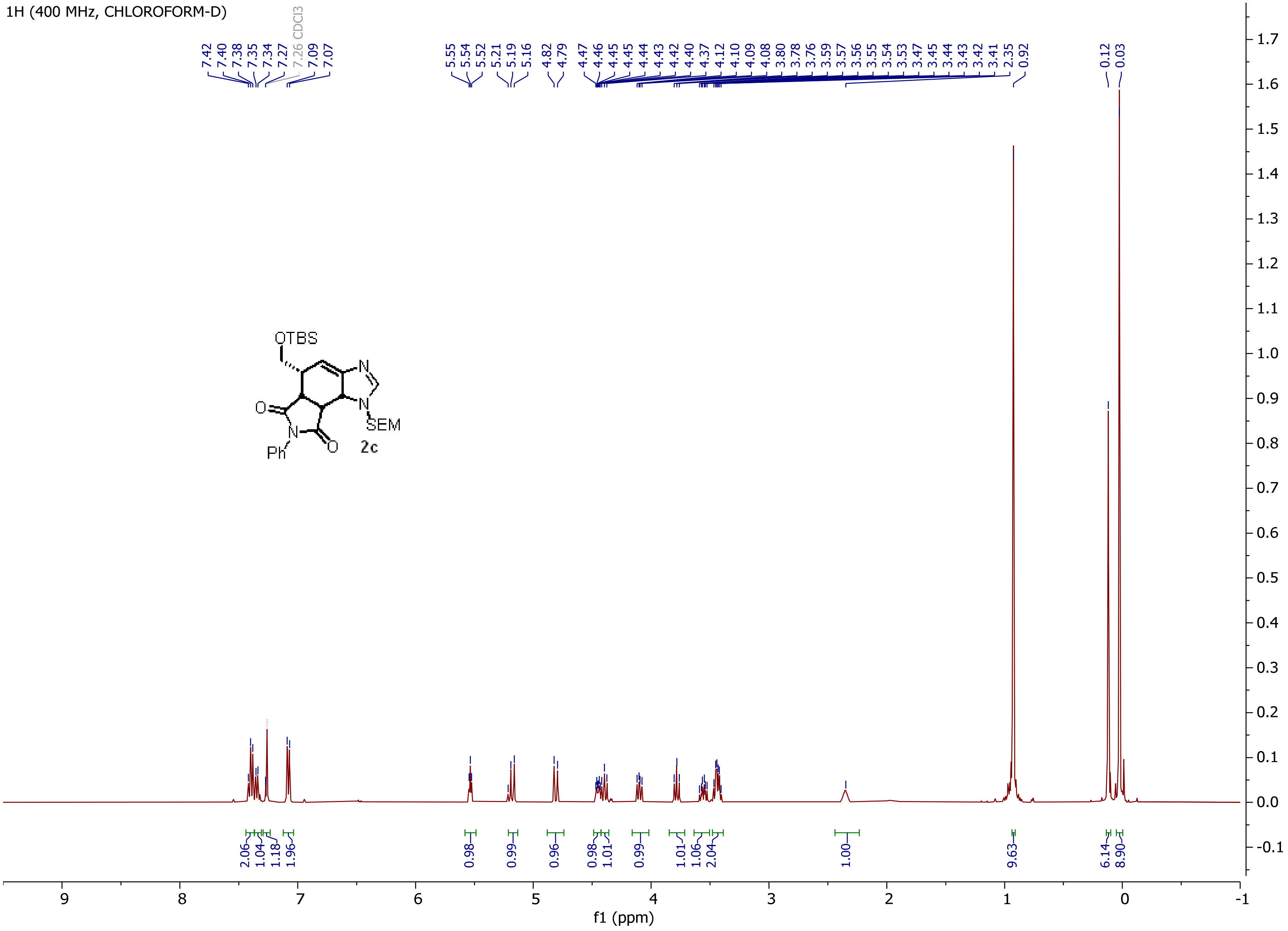
1H (400 MHz, CHLOROFORM-D)
OTBS
N
N
SEM
O
N
O
Ph
2c
f1 (ppm)

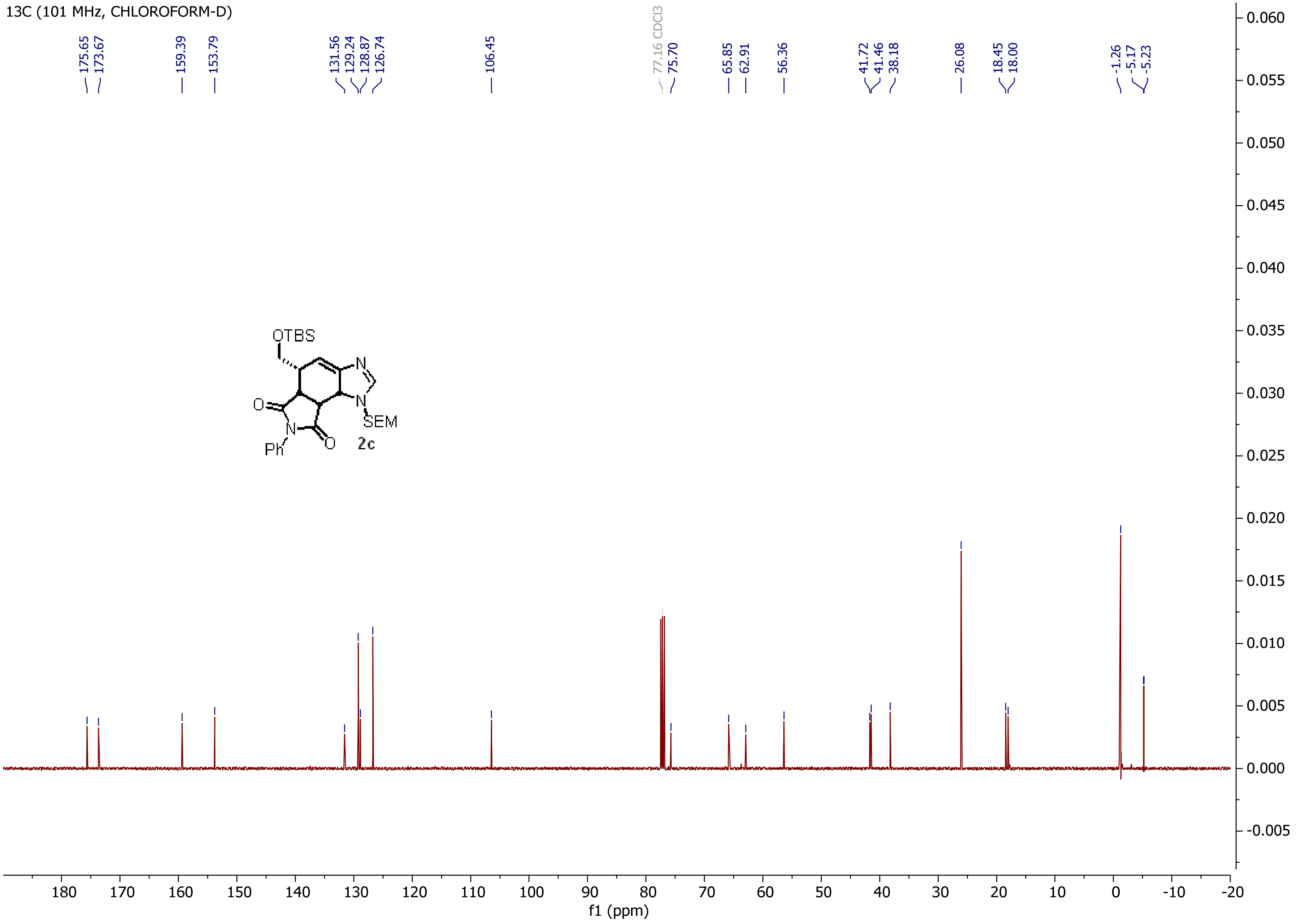
13C (101 MHz, CHLOROFORM-D)
175.65
173.67
159.39
153.79
131.56
129.24
128.87
126.74
106.45
77.16 CDCl3
75.70
65.85
62.91
56.36
41.72
41.46
38.18
26.08
18.45
18.00
-1.26
-5.17
-5.23
OTBS
N
O
N
SEM
Ph
O
2c
f1 (ppm)

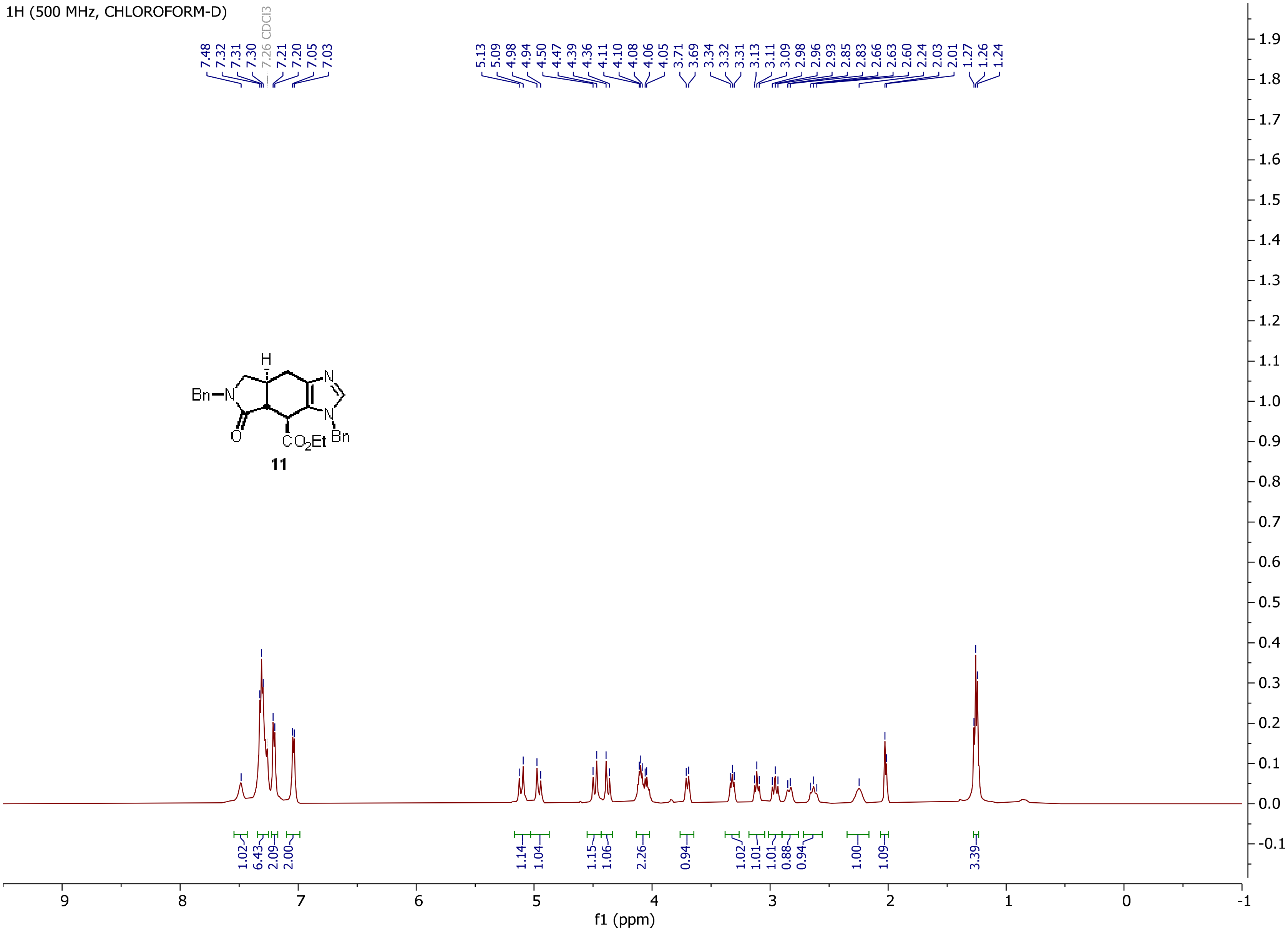
1H (500 MHz, CHLOROFORM-D)
7.48
7.32
7.31
7.30
7.26 CDCl3
7.21
7.20
7.05
7.03
5.13
5.09
4.98
4.94
4.50
4.47
4.39
4.36
4.11
4.10
4.08
4.06
4.05
3.71
3.69
3.34
3.32
3.31
3.13
3.11
3.09
2.98
2.96
2.93
2.85
2.83
2.66
2.63
2.60
2.24
2.03
2.01
1.27
1.26
1.24
1.02
6.43
2.09
2.00
1.14
1.04
1.15
1.06
2.26
0.94
1.02
1.01
1.01
0.88
0.94
1.00
1.09
3.39
Bn
N
O
H
N
N
CO2Et
Bn
11
f1 (ppm)

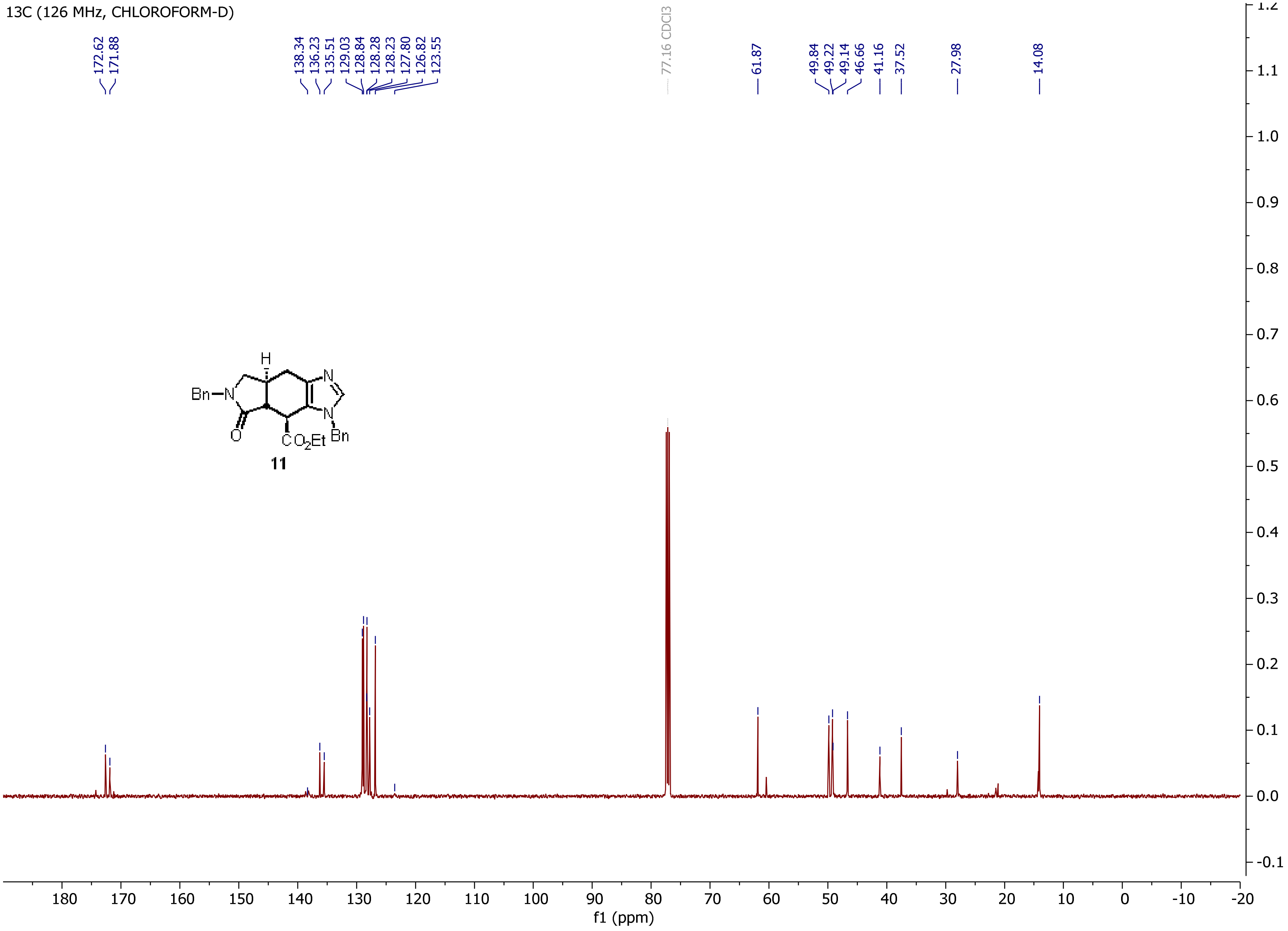
13C (126 MHz, CHLOROFORM-D)
172.62
171.88
138.34
136.23
135.51
129.03
128.84
128.28
128.23
127.80
126.82
123.55
77.16 CDCl3
61.87
49.84
49.22
49.14
46.66
41.16
37.52
27.98
14.08
H
Bn
N
O
N
N
Bn
CO2Et
11
f1 (ppm)

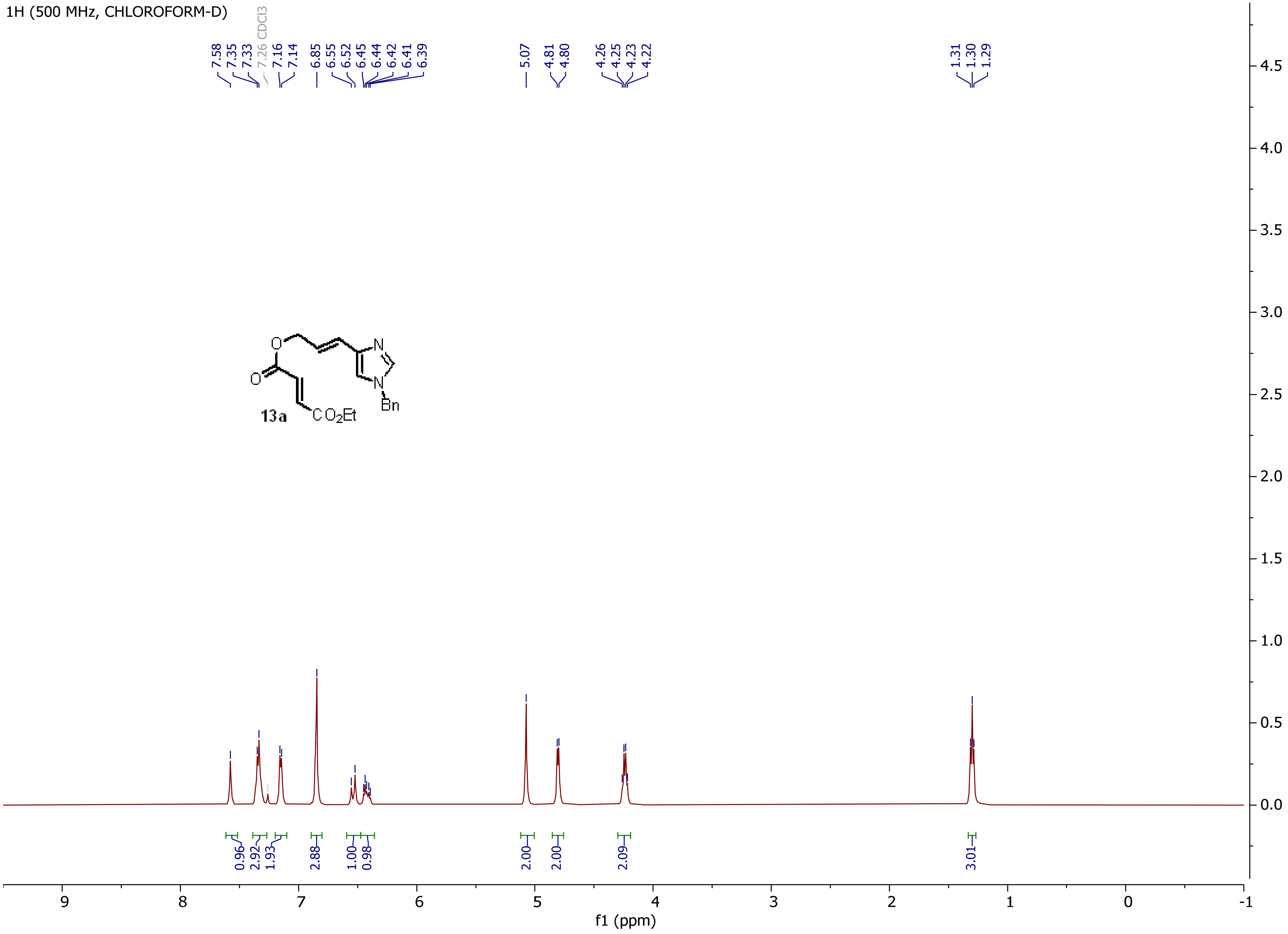
1H (500 MHz, CHLOROFORM-D)
7.58 7.35 7.33 7.26 CDCl3 7.16 7.14 6.85 6.55 6.52 6.45 6.44 6.42 6.41 6.39
5.07 4.81 4.80 4.26 4.25 4.23 4.22
1.31 1.30 1.29
13a
CO2Et
Bn
0.96 2.92 1.93 2.88 1.00 0.98 2.00 2.00 2.09 3.01
f1 (ppm)

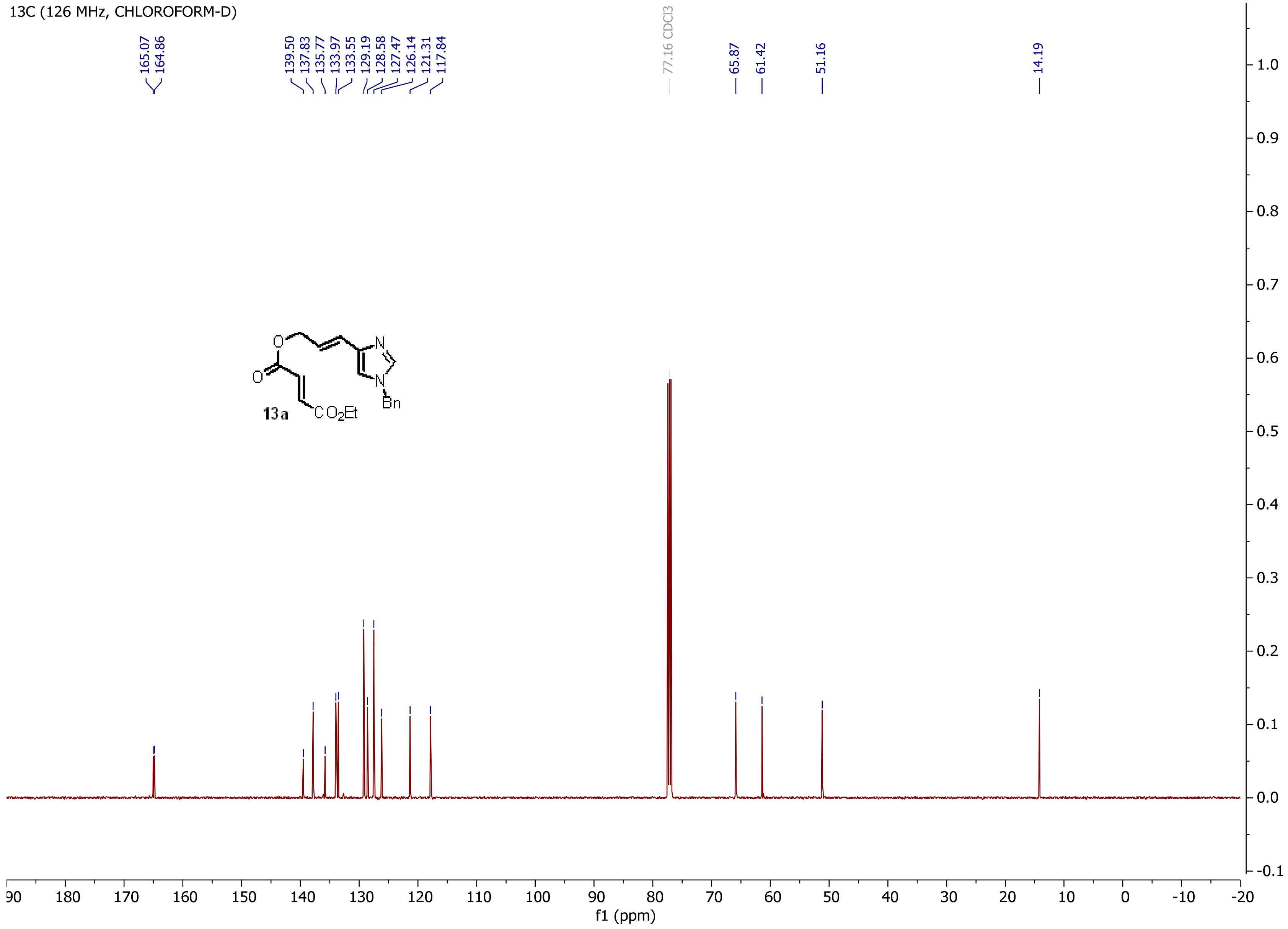
13C (126 MHz, CHLOROFORM-D)
165.07
164.86
139.50
137.83
135.77
133.97
133.55
129.19
128.58
127.47
126.14
121.31
117.84
77.16 CDCl3
65.87
61.42
51.16
14.19
13a
CO2Et
Bn
f1 (ppm)

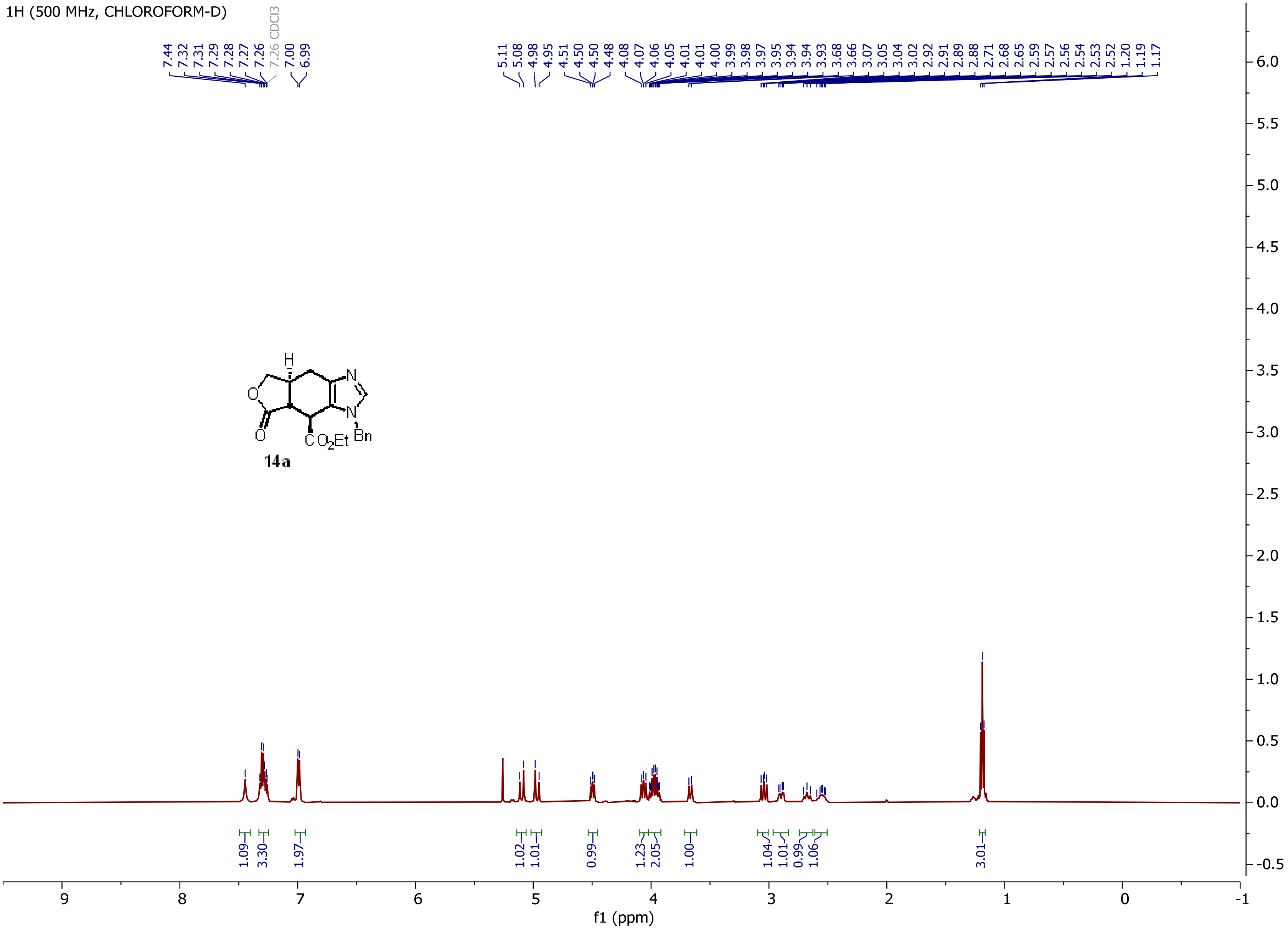
1H (500 MHz, CHLOROFORM-D)
7.44 7.32 7.31 7.29 7.28 7.27 7.26 7.26 CDCl3 7.00 6.99
5.11 5.08 4.98 4.95 4.51 4.50 4.50 4.48 4.08 4.07 4.06 4.05 4.01 4.01 4.00 3.99 3.98 3.97 3.95 3.94 3.93 3.68 3.66 3.07 3.05 3.04 3.02 2.92 2.91 2.89 2.88 2.71 2.68 2.65 2.59 2.57 2.56 2.54 2.53 2.52 1.20 1.19 1.17
1.09 3.30 1.97 1.02 1.01 0.99 1.23 2.05 1.00 1.04 1.01 0.99 1.06 3.01
H
O
O
N
N
Bn
CO2Et
14a
f1 (ppm)

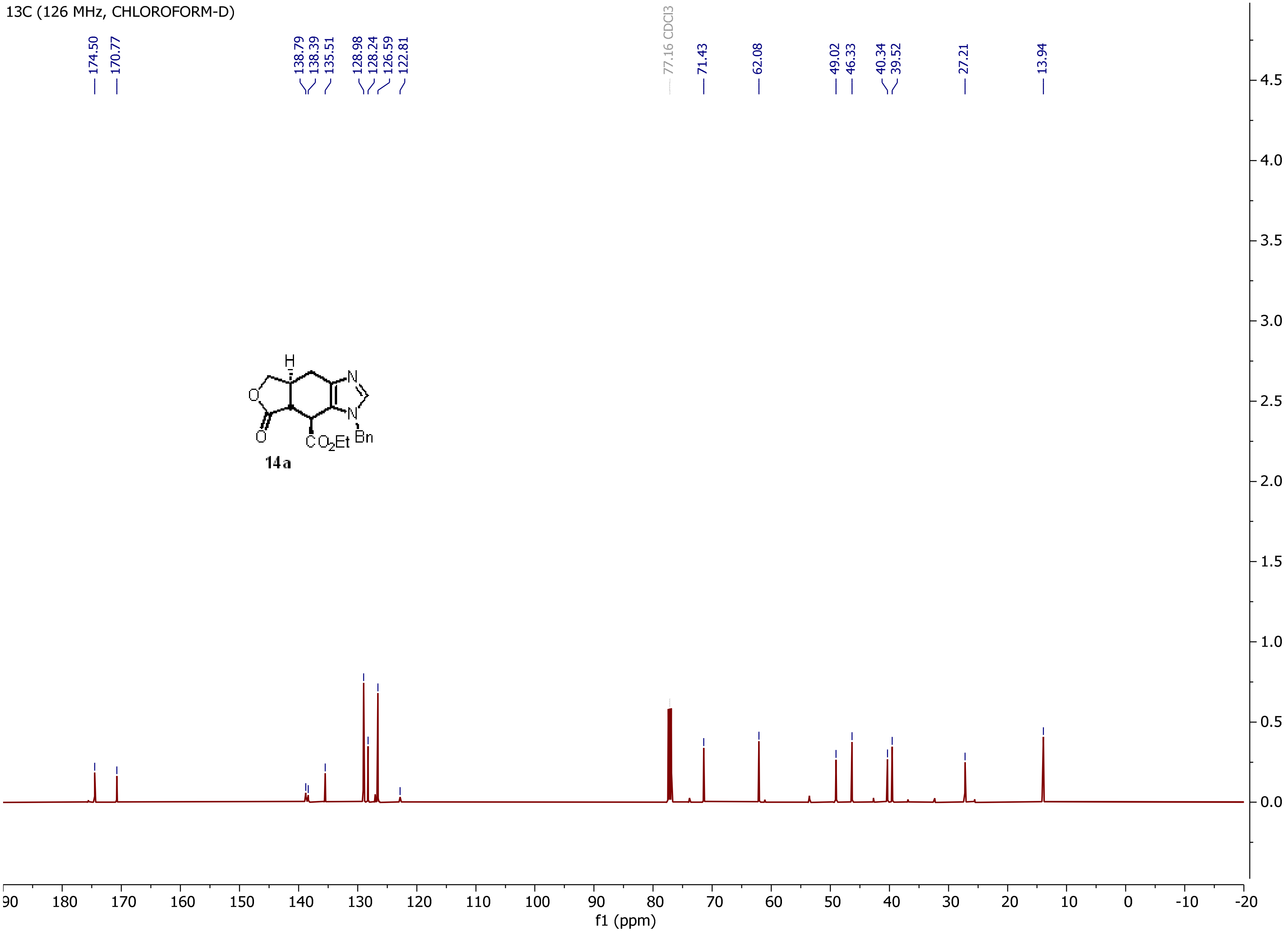
13C (126 MHz, CHLOROFORM-D)
174.50
170.77
138.79
138.39
135.51
128.98
128.24
126.59
122.81
77.16 CDCl3
71.43
62.08
49.02
46.33
40.34
39.52
27.21
13.94
f1 (ppm)
H
O
N
N
O
CO2Et
Bn

14a

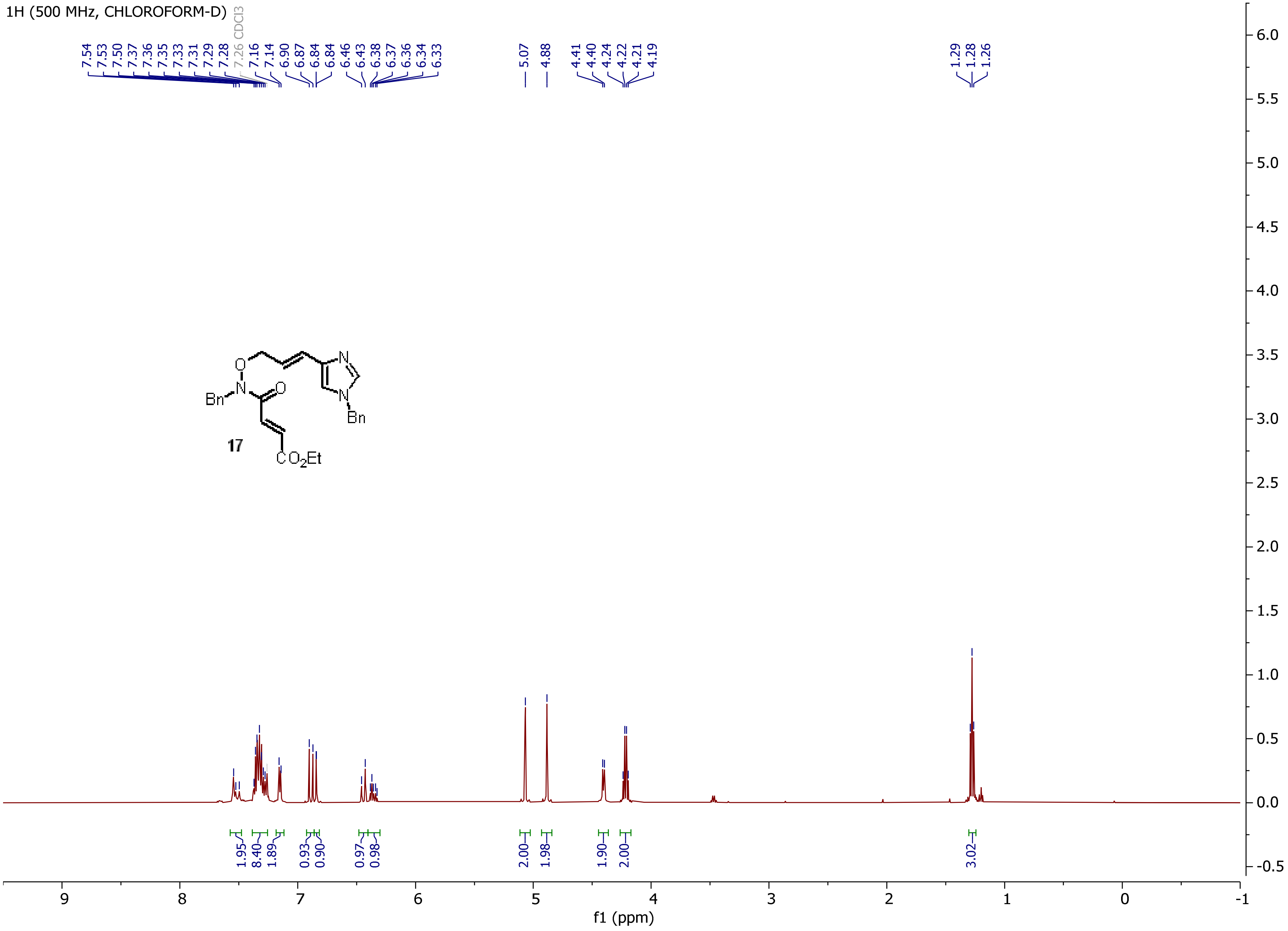
1H (500 MHz, CHLOROFORM-D)
7.54 7.53 7.50 7.37 7.36 7.35 7.33 7.31 7.29 7.28 7.26 CDCl3 7.16 7.14 6.90 6.87 6.84 6.84 6.46 6.43 6.38 6.37 6.36 6.34 6.33
5.07 4.88
4.41 4.40 4.24 4.22 4.21 4.19
1.29 1.28 1.26
1.95 8.40 1.89 0.93 0.90 0.97 0.98 2.00 1.98 1.90 2.00 3.02
17
Bn
N
O
CO2Et
f1 (ppm)

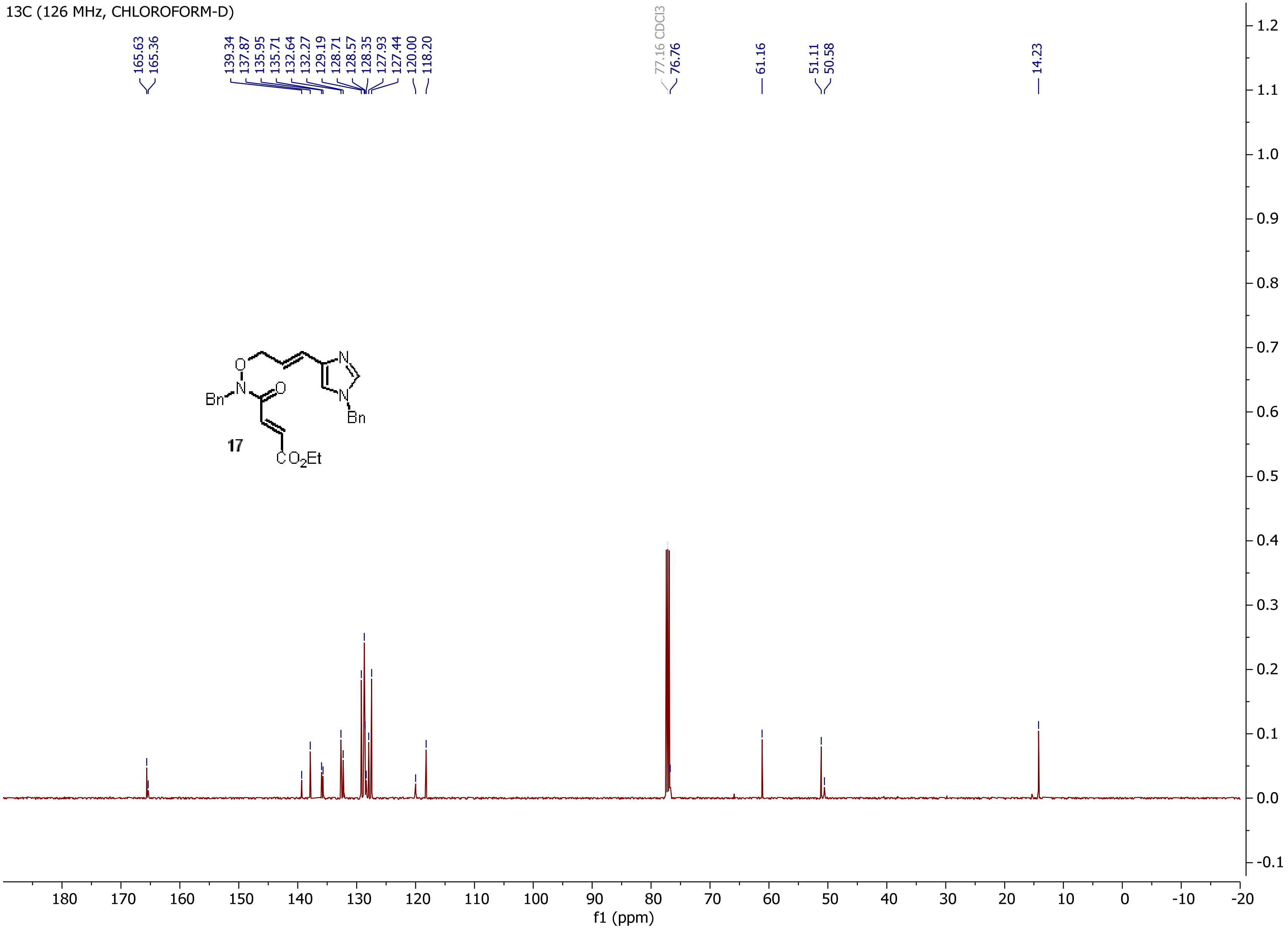
13C (126 MHz, CHLOROFORM-D)
165.63
165.36
139.34
137.87
135.95
135.71
132.64
132.27
129.19
128.71
128.57
128.35
127.93
127.44
120.00
118.20
77.16 CDCl3
76.76
61.16
51.11
50.58
14.23
17
f1 (ppm)

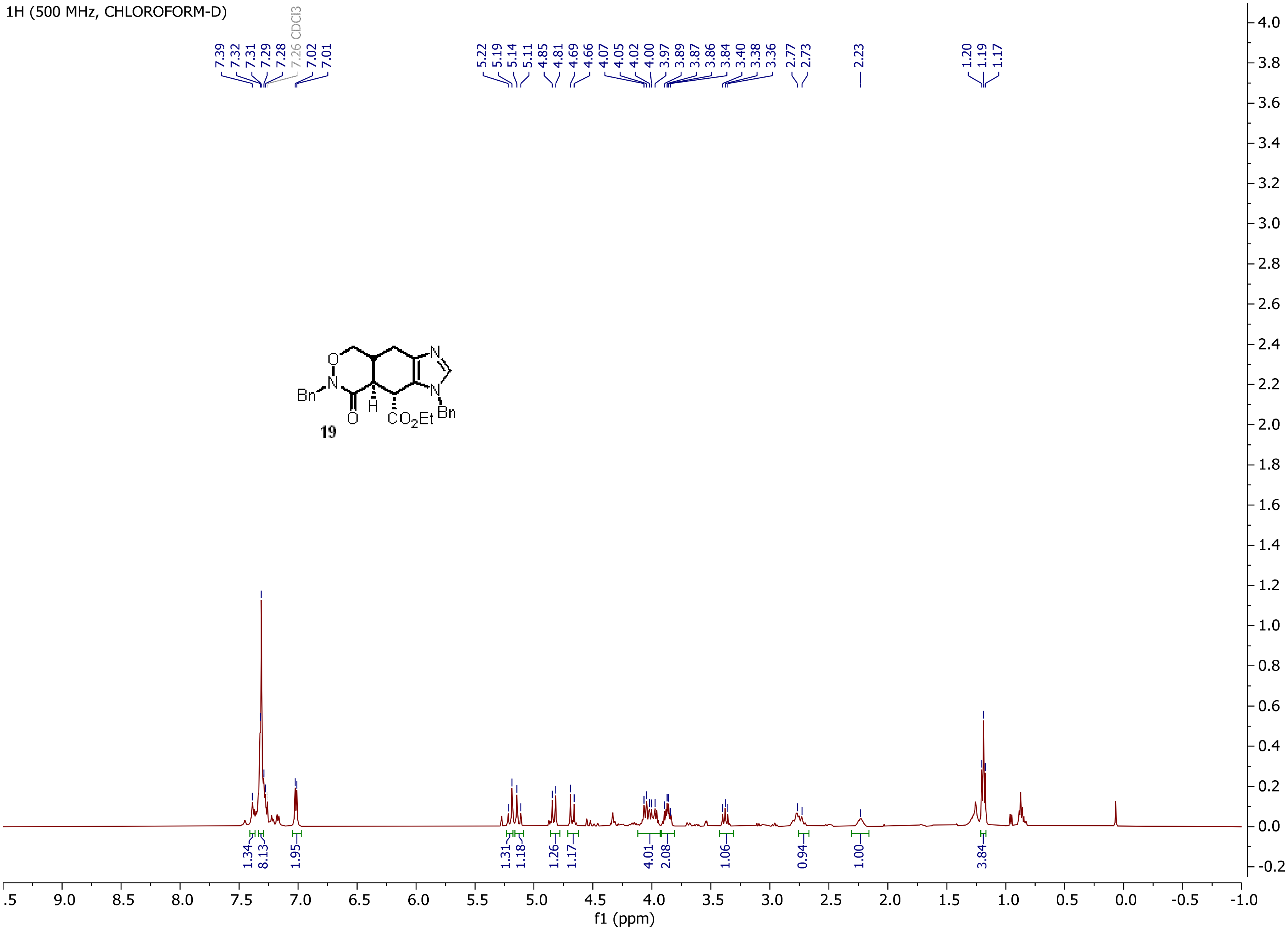
1H (500 MHz, CHLOROFORM-D)
7.39 7.32 7.31 7.29 7.28 7.26 CDCl3 7.02 7.01
5.22 5.19 5.14 5.11 4.85 4.81 4.69 4.66 4.07 4.05 4.02 4.00 3.97 3.89 3.87 3.86 3.84 3.40 3.38 3.36 2.77 2.73
2.23
1.20 1.19 1.17
1.34 8.13 1.95 1.31 1.18 1.26 1.17 4.01 2.08 1.06 0.94 1.00 3.84
Bn
N
O
O
H
CO2Et
N
N
Bn
19
f1 (ppm)

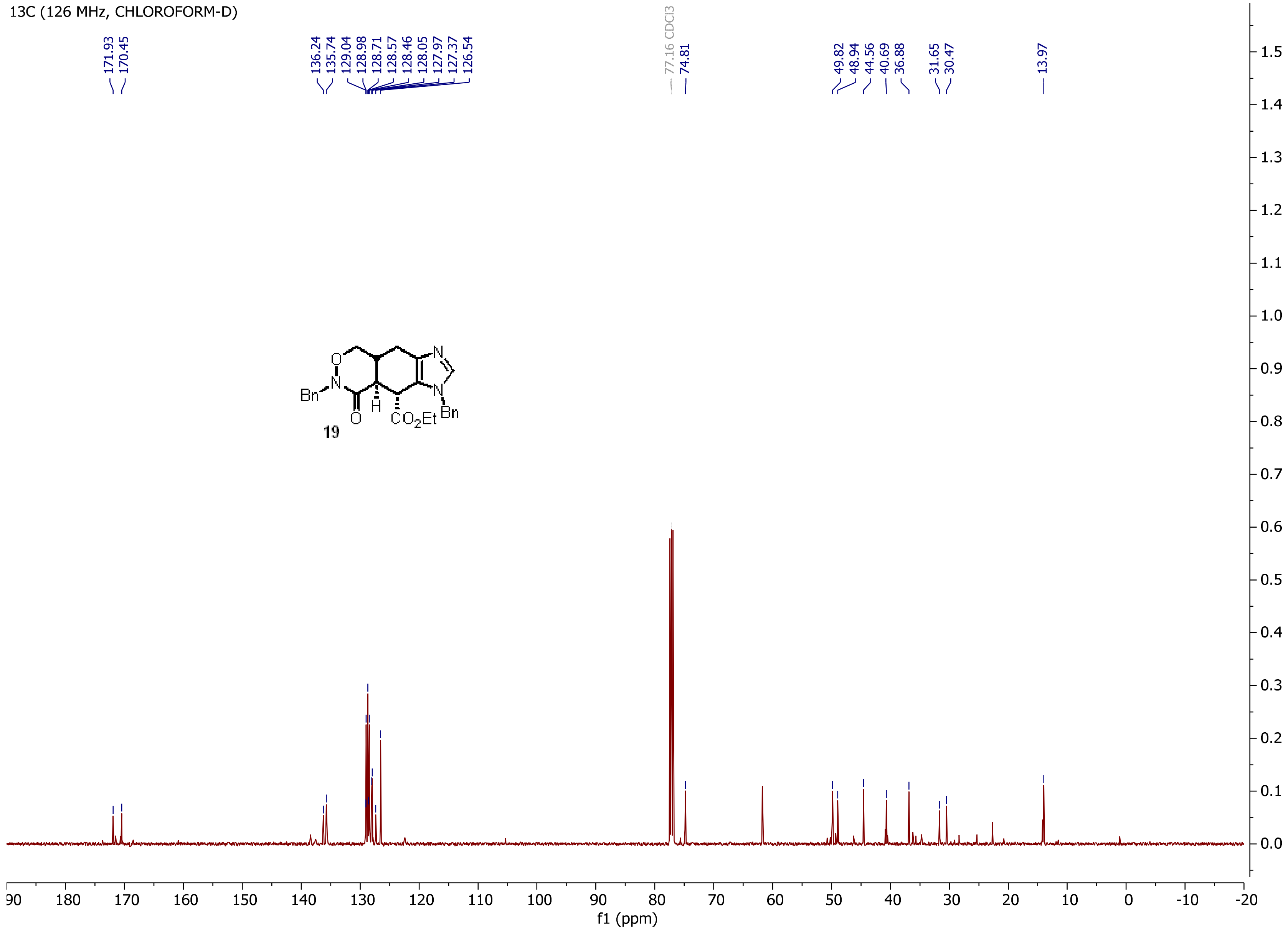

13C (126 MHz, CHLOROFORM-D)
171.93
170.45
136.24
135.74
129.04
128.98
128.71
128.57
128.46
128.05
127.97
127.37
126.54
77.16 CDCl3
74.81
49.82
48.94
44.56
40.69
36.88
31.65
30.47
13.97
19
f1 (ppm)

Computational Data

Cartesian Coordinates for Calculated Gas Phase Endo Transition States

Entry 1 – **2a-TS** Bn (intermolecular)

Atom	X	Y	Z
C	-0.54716	-0.59235	1.36427
C	0.29478	0.30423	2.01652
C	1.66604	0.25432	1.78749
N	2.57249	1.24918	2.13327
C	3.66911	0.93703	1.5089
N	3.59208	-0.22937	0.79418
C	2.28575	-0.65764	0.8837
H	2.05198	-1.69857	0.70852
C	1.16691	-0.15436	-0.94927
C	1.616	1.25562	-0.96198
O	2.73045	1.69311	-1.1617
N	0.48196	2.0329	-0.6568
C	-0.67026	1.23358	-0.62168
O	-1.80642	1.65306	-0.53614
C	-0.20742	-0.17624	-0.68689
H	-0.90369	-0.94112	-1.00531
C	0.49403	3.44591	-0.49717
C	-0.43458	4.23215	-1.17748
C	-0.41269	5.61245	-1.00573
C	0.53574	6.20343	-0.17485
C	1.45934	5.40725	0.49824
C	1.43939	4.02477	0.34782
H	2.13103	3.38716	0.8884
H	2.19479	5.86213	1.15436
H	0.55201	7.28153	-0.04885
H	-1.13796	6.22731	-1.52936
H	-1.16822	3.7606	-1.8206
H	1.71638	-0.90193	-1.50846
C	4.55736	-0.67514	-0.20367
C	4.52276	-2.17388	-0.38221
C	4.71922	-3.02275	0.71105
C	4.68347	-4.40216	0.54454
C	4.45241	-4.94834	-0.71824
C	4.25446	-4.10958	-1.80974
C	4.28577	-2.72605	-1.63981
H	4.13011	-2.06863	-2.49193
H	4.07127	-4.52861	-2.79412

H	4.42528	-6.02569	-0.84742
H	4.83746	-5.05385	1.39891
H	4.89317	-2.59376	1.69511
H	5.54481	-0.34643	0.13773
H	4.33592	-0.16272	-1.14678
H	4.58364	1.51781	1.52065
H	-0.10584	1.18939	2.50637
C	-2.04126	-0.51483	1.55051
O	-2.69322	-1.23723	0.53408
Si	-4.13398	-0.79227	-0.21118
C	-4.80537	0.80263	0.51244
H	-5.00416	0.72635	1.5869
H	-5.74791	1.06392	0.01862
H	-4.1003	1.62194	0.34505
C	-3.79879	-0.61028	-2.04714
H	-3.25803	-1.47921	-2.43743
H	-4.72666	-0.50892	-2.6208
H	-3.18926	0.28182	-2.21904
C	-5.32165	-2.23421	0.11326
C	-6.72693	-1.90395	-0.40548
H	-6.72508	-1.68676	-1.48063
H	-7.40379	-2.75455	-0.24493
H	-7.15766	-1.03931	0.11268
C	-5.38403	-2.49951	1.62349
H	-4.39469	-2.75123	2.0218
H	-5.76086	-1.62884	2.17385
H	-6.05749	-3.34072	1.8389
C	-4.79977	-3.49256	-0.594
H	-4.79221	-3.36903	-1.68325
H	-3.78068	-3.73721	-0.27281
H	-5.44057	-4.35526	-0.36395
H	-2.29615	-0.95396	2.52756
H	-2.3503	0.53792	1.56409
H	-0.17875	-1.59757	1.16687

Entry 2 – **2c-TS** SEM (intermolecular)

Atom	X	Y	Z
C	-0.49177	-0.54184	1.08254
C	0.07494	0.52462	1.77365
C	1.33533	0.98865	1.40928
N	1.89501	2.19596	1.81184
C	2.93864	2.35444	1.05327
N	3.1509	1.32016	0.18046
C	4.07744	1.29032	-0.94048
H	3.53272	1.53607	-1.85583
H	4.84283	2.05555	-0.75062
O	4.62024	0.02198	-1.0993
C	5.58989	-0.31765	-0.11106
H	6.47032	0.32841	-0.24931
H	5.18173	-0.1193	0.89079
C	5.93375	-1.78896	-0.27067
H	6.72203	-2.04189	0.45034
H	6.35786	-1.95242	-1.26906
Si	4.47595	-2.9733	0.01631
C	3.67619	-2.55925	1.67536
H	3.34185	-1.51938	1.75493
H	2.80504	-3.19966	1.85328
H	4.38463	-2.73729	2.49225
C	3.22032	-2.8879	-1.38773
H	2.31323	-3.45067	-1.14062
H	3.64228	-3.32495	-2.29935
H	2.94522	-1.85646	-1.62459
C	5.16207	-4.72609	0.10133
H	5.87443	-4.83666	0.92564
H	4.35894	-5.45446	0.2555
H	5.67885	-4.99468	-0.82615
C	2.08903	0.45418	0.32506
H	2.1956	-0.57652	0.01808
C	0.62604	0.70494	-1.30695
H	1.30088	0.25134	-2.02246
C	0.58267	2.17749	-1.16305
O	1.45357	2.9854	-1.41017
N	-0.68691	2.48281	-0.63453
C	-1.49418	1.33626	-0.59291
O	-2.67862	1.32709	-0.32729
C	-0.61004	0.18528	-0.91168
H	-1.05419	-0.73696	-1.26318

C	-1.11944	3.79074	-0.28115
C	-0.31042	4.5753	0.53889
C	-0.72814	5.85793	0.87768
C	-1.94765	6.34749	0.4161
C	-2.7535	5.54925	-0.39184
C	-2.34122	4.27022	-0.75101
H	-2.95979	3.63941	-1.37846
H	-3.70693	5.92368	-0.75103
H	-2.27153	7.34702	0.6889
H	-0.10049	6.4728	1.5151
H	0.6213	4.16648	0.91539
H	3.601	3.21159	1.0699
H	-0.53029	1.15215	2.42463
H	0.16736	-1.3215	0.70234
C	-1.88081	-1.02191	1.41909
H	-1.82889	-1.62407	2.33951
H	-2.52431	-0.15624	1.62013
O	-2.38629	-1.81696	0.3743
Si	-3.97919	-1.82294	-0.16759
C	-5.04034	-0.65231	0.84313
H	-6.07507	-0.69012	0.4848
H	-5.04911	-0.90617	1.90861
H	-4.68412	0.37583	0.73201
C	-3.97174	-1.33236	-1.97732
H	-4.95047	-1.48895	-2.44388
H	-3.23535	-1.91426	-2.54204
H	-3.71581	-0.27222	-2.06538
C	-4.56129	-3.61499	0.04115
C	-6.04563	-3.74724	-0.32225
H	-6.2424	-3.43524	-1.35514
H	-6.37081	-4.79266	-0.22878
H	-6.68042	-3.14584	0.33883
C	-4.3527	-4.04039	1.50079
H	-4.66479	-5.08399	1.64567
H	-3.29812	-3.96271	1.78862
H	-4.94028	-3.42362	2.19153
C	-3.72992	-4.5289	-0.8697
H	-4.00961	-5.58046	-0.71561
H	-3.89105	-4.29718	-1.92899
H	-2.65835	-4.43292	-0.66015

Entry 3 – **2b-TS** DMAS (intermolecular)

Atom	X	Y	Z
C	0.41251	-2.24291	-0.00071
C	0.04525	-1.36056	-1.00383
C	-1.28743	-0.98168	-1.1371
N	-1.75561	0.02475	-1.98063
C	-2.99776	0.20401	-1.67081
N	-3.41718	-0.61428	-0.63448
S	-4.99889	-0.94189	-0.11922
O	-5.65223	-1.79641	-1.08438
O	-4.82022	-1.38372	1.25098
N	-5.64902	0.56339	-0.25482
C	-5.30759	1.47891	0.84311
H	-5.86962	1.23594	1.75225
H	-5.5617	2.48808	0.51235
H	-4.23385	1.44226	1.03678
C	-7.05508	0.60568	-0.67723
H	-7.73498	0.3363	0.14005
H	-7.26183	1.63105	-0.99013
H	-7.20646	-0.06669	-1.51958
C	-2.30861	-1.35458	-0.21653
C	-1.53241	-0.60307	1.57756
C	-1.28626	0.79743	1.11498
O	-2.10166	1.63671	0.79329
N	0.0977	0.96694	1.08326
C	0.75104	-0.13531	1.67907
O	1.92085	-0.16738	1.9886
C	-0.28375	-1.17638	1.84209
H	-0.10434	-2.0109	2.50766
C	0.76276	2.09139	0.52239
C	0.41361	2.50774	-0.76257
C	1.08888	3.58044	-1.33378
C	2.11162	4.22162	-0.6371
C	2.45368	3.79329	0.64251
C	1.77429	2.73193	1.23402
H	2.03228	2.38651	2.22851
H	3.25174	4.28631	1.18884
H	2.64303	5.05134	-1.09271
H	0.82278	3.90691	-2.33412
H	-0.36603	1.98017	-1.30488
H	-2.45845	-0.84127	2.08928
H	-2.47596	-2.32457	0.23187

H	-3.67508	0.92553	-2.10762
H	0.80862	-0.82075	-1.55613
C	1.86395	-2.58866	0.21987
O	2.67565	-1.63929	-0.41126
Si	4.3432	-1.5662	-0.20375
C	4.77767	-2.12827	1.53415
H	4.57859	-3.19523	1.68279
H	4.19822	-1.56055	2.2698
H	5.84189	-1.96243	1.7361
C	5.11366	-2.72893	-1.46592
H	6.20347	-2.77003	-1.36594
H	4.72916	-3.74713	-1.33945
H	4.88035	-2.41087	-2.48764
C	4.85102	0.23213	-0.51733
C	3.82737	0.89895	-1.4475
H	2.83605	0.93123	-0.98321
H	4.13117	1.93275	-1.66326
H	3.73645	0.36935	-2.40445
C	6.24165	0.27505	-1.16745
H	6.55949	1.31751	-1.30443
H	6.24877	-0.20673	-2.15162
H	7.00043	-0.2188	-0.54643
C	4.89602	1.00371	0.8097
H	5.11925	2.06284	0.61701
H	3.93785	0.95007	1.33567
H	5.67606	0.61526	1.47555
H	2.07344	-2.63073	1.29869
H	2.04492	-3.59697	-0.18993
H	-0.29535	-3.00511	0.31753

Entry 4 – **10-TS** N-linked (intramolecular)

Atom	X	Y	Z
C	-0.81258	-0.24249	1.42307
C	-0.19643	0.83457	2.06325
C	1.17871	0.99656	1.89681
N	1.88307	2.15204	2.21222
C	3.06131	1.97553	1.69522
N	3.21129	0.76769	1.05858
C	4.4071	0.34031	0.3203
C	4.34807	-1.12654	-0.02193
C	3.83267	-1.53041	-1.25553
C	3.73472	-2.8865	-1.55933
C	4.14978	-3.84236	-0.63492
C	4.6636	-3.44244	0.59711
C	4.76082	-2.08799	0.90211
H	5.15846	-1.77204	1.86384
H	4.9902	-4.18491	1.31861
H	4.07432	-4.89863	-0.87405
H	3.33327	-3.19576	-2.51934
H	3.49612	-0.77109	-1.95764
H	4.47003	0.93742	-0.59222
H	5.27447	0.55356	0.95318
C	2.00836	0.12902	1.14311
H	1.93262	-0.92596	0.93607
C	0.60538	0.1419	-0.92847
C	1.32121	1.35221	-1.30095
O	2.43339	1.38232	-1.80807
O	0.6269	2.48033	-1.02578
C	1.18881	3.70664	-1.50567
H	1.58316	3.54299	-2.51195
H	0.33874	4.39028	-1.55961
C	2.2653	4.2465	-0.58297
H	2.57561	5.24173	-0.91626
H	3.13416	3.58437	-0.60146
H	1.8909	4.31279	0.44188
H	1.05335	-0.7841	-1.27384
C	-0.70932	0.16189	-0.44983
C	-1.7183	-0.90617	-0.84367
O	-1.81095	-1.38042	-1.9603
N	-2.55318	-1.21314	0.19601
C	-2.291	-0.5378	1.45548
H	-2.55716	-1.21	2.28102

H	-2.88707	0.38077	1.56144
C	-3.7682	-1.97748	-0.00577
C	-4.99337	-1.08902	-0.02452
C	-5.15183	-0.16365	-1.06095
C	-6.25127	0.68638	-1.08021
C	-7.20379	0.62186	-0.06257
C	-7.05046	-0.2947	0.97207
C	-5.94555	-1.14526	0.99041
H	-5.82376	-1.86133	1.80012
H	-7.78787	-0.34818	1.76717
H	-8.06297	1.28528	-0.07925
H	-6.37013	1.39853	-1.89109
H	-4.40711	-0.1253	-1.85339
H	-3.85435	-2.72696	0.79056
H	-3.64673	-2.49725	-0.95965
H	-1.17837	1.14624	-0.43691
H	3.8789	2.68553	1.73475
H	-0.7698	1.67333	2.44949
H	-0.22447	-1.15881	1.36426

Entry 5 – **18-TS** NO-linked (intramolecular)

Atom	X	Y	Z
C	-1.29519	-0.71649	1.65805
C	-0.22804	-1.60387	1.72396
C	0.85127	-1.38494	0.86866
C	2.02274	-2.32315	0.77941
H	2.90896	-1.89125	1.257
H	1.79332	-3.28798	1.23893
O	2.35107	-2.67893	-0.58935
N	2.38208	-1.59054	-1.44093
C	1.21336	-0.89856	-1.63862
O	1.20008	0.20002	-2.18403
C	0.01882	-1.54411	-1.03968
H	-0.00209	-2.62431	-0.9844
C	-1.18912	-0.83895	-1.11956
C	-2.48268	-1.52652	-0.98156
O	-3.56137	-1.00161	-1.18753
O	-2.34969	-2.80101	-0.57083
C	-3.55744	-3.57023	-0.4727
C	-4.32416	-3.27789	0.80309
H	-3.66693	-3.34703	1.67295
H	-4.74336	-2.27088	0.77033
H	-5.1415	-3.99769	0.91131
H	-3.21487	-4.60663	-0.50135
H	-4.17079	-3.37011	-1.35536
H	-1.21523	0.07359	-1.70594
C	-1.36036	0.34675	0.70735
N	-2.62616	0.86307	0.84411
C	-3.28723	0.04732	1.72425
N	-2.54307	-0.88486	2.2439
H	-4.33798	0.20257	1.93933
C	-3.2107	1.87583	-0.03073
C	-2.25619	3.02968	-0.2238
C	-1.52639	3.14988	-1.40594
C	-0.5989	4.17872	-1.5613
C	-0.40099	5.09629	-0.53458
C	-1.12741	4.98182	0.65068
C	-2.04649	3.9506	0.80561
H	-2.6054	3.85102	1.73322
H	-0.97518	5.69647	1.45357
H	0.31898	5.89986	-0.6545
H	-0.03159	4.25711	-2.48304

H	-1.68026	2.43345	-2.2092
H	-4.14092	2.20737	0.44085
H	-3.45849	1.39967	-0.98526
H	-0.56457	0.97937	0.33508
C	3.69054	-0.99894	-1.6669
C	4.21563	-0.26468	-0.45272
C	3.59665	0.92068	-0.03902
C	4.021	1.56406	1.11907
C	5.07032	1.03548	1.87044
C	5.69736	-0.13615	1.4565
C	5.26942	-0.78338	0.2984
H	5.74848	-1.7063	-0.01968
H	6.51712	-0.54961	2.03606
H	5.40003	1.53875	2.77407
H	3.53547	2.48243	1.43508
H	2.78067	1.32513	-0.63408
H	4.36837	-1.80508	-1.96025
H	3.56064	-0.31903	-2.51164
H	1.11318	-0.34199	0.69172
H	-0.3617	-2.54594	2.24924

Entry 6 – **14a-TS** O-linked (intramolecular)

Atom	X	Y	Z
C	-1.85636	-1.50212	1.40908
C	-1.94477	-0.22944	1.9752
C	-0.85595	0.62798	1.82519
N	-0.89472	1.997	2.05607
C	0.22125	2.44901	1.56903
N	1.0271	1.47407	1.03367
C	2.30351	1.71642	0.34662
C	3.0214	0.42652	0.04315
C	3.83445	-0.17632	1.00455
C	4.45943	-1.39025	0.73405
C	4.27253	-2.00855	-0.50065
C	3.46205	-1.41072	-1.46302
C	2.83696	-0.1953	-1.1936
H	2.18604	0.27572	-1.92636
H	3.31436	-1.89108	-2.42515
H	4.75942	-2.95539	-0.71234
H	5.09325	-1.85204	1.48462
H	3.97627	0.308	1.96801
H	2.08588	2.25372	-0.57925
H	2.91123	2.35137	0.99916
C	0.34744	0.29762	1.14919
H	0.85593	-0.6445	1.02184
C	-0.74006	-0.50979	-0.93556
C	-0.81604	0.88247	-1.35624
O	0.11191	1.52456	-1.82431
O	-2.04298	1.41847	-1.16907
C	-2.25265	2.73356	-1.69859
H	-1.78752	2.79178	-2.68616
H	-3.33664	2.80913	-1.8074
C	-1.71793	3.81802	-0.78238
H	-2.02428	4.80049	-1.15469
H	-2.10058	3.6847	0.23281
H	-0.6267	3.77751	-0.75824
H	0.16426	-1.03763	-1.21847
C	-1.86161	-1.21366	-0.48641
C	-2.00916	-2.6934	-0.752
O	-1.56558	-3.29187	-1.6897
O	-2.735	-3.32658	0.20618
C	-2.96671	-2.51622	1.36416
H	-2.96603	-3.19717	2.2187

H	-3.94867	-2.03282	1.29279
H	-2.81163	-0.68481	-0.55747
H	0.52894	3.48789	1.56072
H	-2.89903	0.20202	2.26651
H	-0.87456	-1.97588	1.45679
